# A novel copper-chelating strategy for fluorescent proteins to image dynamic copper fluctuations on live cell surfaces†
†Electronic supplementary information (ESI) available. See DOI: 10.1039/c4sc03027c


**DOI:** 10.1039/c4sc03027c

**Published:** 2014-11-19

**Authors:** Yoon-Aa Choi, Joo Oak Keem, Cha Yeon Kim, Hye Ryeon Yoon, Won Do Heo, Bong Hyun Chung, Yongwon Jung

**Affiliations:** a BioNano Health Guard Research Center , 125 Gwahak-ro, Yuseong-gu , Daejeon , 305-806 , Republic of Korea . Email: chungbh@kribb.re.kr ; Fax: +82-42-860-4209 ; Tel: +82-42-860-4442; b Graduate School of Nanoscience and Technology , Korea Advanced Institute of Science and Technology (KAIST) , Republic of Korea; c Department of Chemistry , KAIST , 291 Daehak-ro, Yuseong-gu , Daejeon , 305-701 , Republic of Korea . Email: ywjung@kaist.ac.kr ; Fax: +82-42-350-2810 ; Tel: +82-42-350-2817; d Department of Biological Sciences , KAIST , Republic of Korea

## Abstract

Copper is indispensable in most aerobic organisms although it is toxic if unregulated as illustrated in many neurodegenerative diseases. To elucidate the mechanisms underlying copper release from cells, a membrane-targeting reporter which can compete with extracellular copper-binding molecules is highly desirable. However, engineering a reporter protein to provide both high sensitivity and selectivity for copper(ii) has been challenging, likely due to a lack of proper copper(ii)-chelating strategies within proteins. Here, we report a new genetically encoded fluorescent copper(ii) reporter by employing a copper-binding tripeptide derived from human serum albumin (HSA), which is one of the major copper-binding proteins in extracellular environments. Optimized insertion of the tripeptide into the green fluorescent protein leads to rapid fluorescence quenching (up to >85% change) upon copper-binding, while other metal ions have no effect. Furthermore, the high binding affinity of the reporter enables reliable copper detection even in the presence of competing biomolecules such as HSA and amyloid beta peptides. We also demonstrate that our reporter proteins can be used to visualize dynamic copper fluctuations on living HeLa cell surfaces.

## Abstract 2

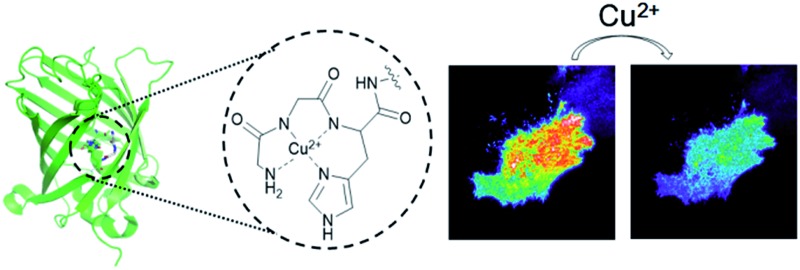
A strong but selective copper-binding tripeptide was employed to develop a highly sensitive and selective copper(ii) protein reporter.

## Introduction

Copper is an essential metal ion in most aerobic organisms, participating in many critical biological processes including energy generation, oxygen transport, cellular metabolism and signal transduction.^1^ However, when unregulated, copper can become toxic by generating reactive oxygen species (ROS) *via* Fenton-type reactions.^2^ The adverse effect of dysregulated copper homeostasis in humans is well illustrated in Menkes and Wilson's diseases, which are related to copper deficiency or overload, respectively.^3^ To address copper transport and trafficking in living systems, a variety of synthetic and genetically encoded probes have been developed with a focus mainly on visualizing intracellular copper,^4^ where Cu^+^ is the major form due to reduction upon cellular uptake and the reducing environment of the cytosol.^5^

In contrast, extracellular copper trafficking has been insufficiently investigated due to a lack of appropriate tools for monitoring in living systems. In the more oxidizing extracellular media, Cu^2+^ is favored over Cu^+^.^6^ This oxidized form of copper has long been linked to many neurodegenerative diseases such as Alzheimer's disease, prion diseases, and Parkinson's disease through its participation in the aggregation of fibrogenic proteins.^6^ The protein–Cu^2+^ interactions are also directly involved in a catalytic cycle of ROS production, which exerts oxidative damage to cells.^7^ Moreover, exogenous copper plays a key role in neuronal signaling pathways by modulating the synaptic plasticity.^8^ Therefore, monitoring dynamic changes in the copper concentration in the extracellular environment will be critical for understanding the mechanisms of Cu^2+^ release and its physiological functions in the brain. In particular, imaging Cu^2+^ fluctuations on specifically targeted cell surfaces will greatly improve our insight into cellular responses to outside copper and copper-based cell-to-cell communication. Although a few cellular targeting strategies have been devised for synthetic metal reporters using organelle-targeting moieties,^9^ they cannot direct the probes to specific cells of interest. Furthermore, mislocalization of small-molecular probes inside cells might lead to controversy in data interpretation.^10^ In comparison, genetically encodable protein reporters have major advantages for the cell-specific and surface-specific detection of target molecules.

Several genetically encoded reporters have been applied for imaging copper in cellular context.^11^ However, they are limited by relatively small responses (15–50%),11b–e low copper selectivity,11a,11e or reversed responses in mammalian cells compared with the response *in vitro* and in living bacteria.11*b* Another challenge posed by imaging the extracellular pool of copper in particular is to provide the necessary binding affinity for Cu^2+^ sensors to compete with copper-binding molecules in the extracellular medium. While extracellular Cu^2+^ is present at micromolar concentrations (10–25 μM in blood plasma, 0.5–2.5 μM in cerebrospinal fluid (CSF), and 30 μM in the synaptic cleft),^6^ the metal ion is rather bound to highly abundant copper-binding proteins or small molecular ligands than present as a free ion.^12^ For example, human serum albumin (HSA), found at ∼600 μM in plasma, binds Cu^2+^ with a picomolar binding affinity.^13^ The HSA concentration in CSF is relatively low (∼3 μM) but represents 35–80% of the total CSF proteins.^14^

Developing protein-based Cu^2+^ sensors with strong but selective copper-binding properties will require potent Cu^2+^-chelating strategies within proteins. Previously reported sensors have employed simple histidines or a metal-chelating unnatural amino acid (3,4-dihydroxy-l-phenylalanine) to bind Cu^2+^ since the metal ion prefers to bind nitrogen or oxygen donors.11a,^15^ Unfortunately, reported binding affinities (*K*_D_ 0.2–100 μM) of these sensors under physiologically relevant conditions have been still insufficient for imaging extracellular Cu^2+^, and it has not been demonstrated whether they could detect changes in copper concentration under conditions where competing copper-binding biomolecules are present.

Here, we report a new genetically encoded fluorescent reporter that binds Cu^2+^ with a *K*_D_ of ∼8 nM while maintaining its selectivity for the metal ion. Green fluorescent protein (GFP) was engineered to contain a natural short but strong Cu^2+^-binding tripeptide, the amino terminal copper- and nickel-binding (ATCUN) motif, which is also the major copper-binding site of albumins in many species including humans.^16^ The location of this copper binding sequence in GFP was carefully optimized to provide maximum fluorescence quenching, strong Cu^2+^ binding, and stable protein folding in cells. The resulting reporter was sensitive enough to detect Cu^2+^ even in the presence of equimolar HSA *in vitro*. Furthermore, our reporter was stably displayed on cell surfaces and showed specific and reversible responses to extracellular Cu^2+^.

## Results and discussion

The ATCUN motif consists of three amino acids (Xaa-Xaa-His) with a free N-terminus, where Xaa is any amino acid that can provide an amide nitrogen. As depicted in the X-ray crystal structure, this motif binds Cu^2+^ by forming a square-planar complex with the α-NH_2_-terminal nitrogen, the two amide nitrogens, and the imidazole nitrogen in histidine.^17^ Although the peptide binds Cu^2+^ (*K*_D_ ∼ 1.18 × 10^–16^ M)^18^ and Ni^2+^ (*K*_D_ ∼ 10^–16^ M)^19^ with similarly high affinities, it is highly context dependent, such that the ATCUN in HSA was reported to bind Ni^2+^ with a nearly five orders of magnitude weaker binding affinity than Cu^2+^.^20^ Considering its small size and strong binding affinity for Cu^2+^, we hypothesized that the ATCUN motif can serve as a powerful Cu^2+^-binding site for protein sensors.

**Fig. 1:**
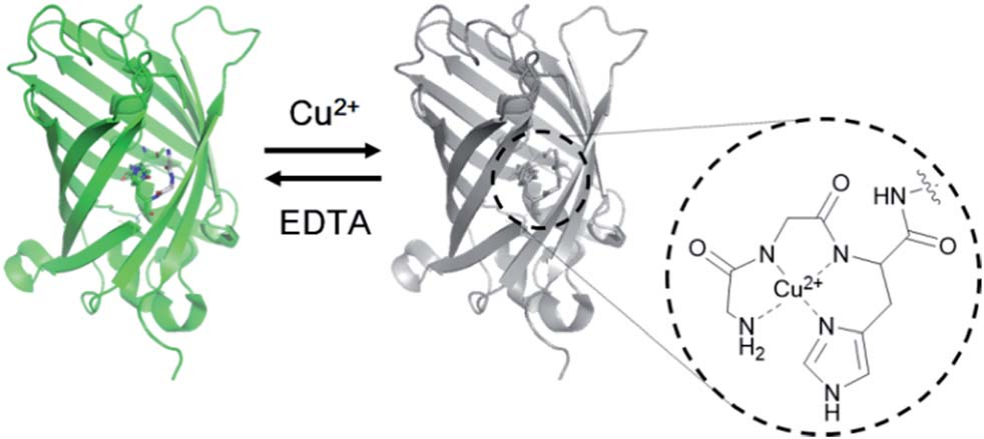
Design of a genetically encoded fluorescent Cu^2+^ reporter using the ATCUN motif. Structure of the Cu^2+^ complex of the ATCUN motif is shown.

To place the ATCUN motif in close proximity of the GFP chromophore, thereby inducing maximum fluorescence quenching by Cu^2+^ binding, the protein was circularly permuted to generate new N- and C-termini at the beginning of β-strand 7. Circular permutations on β-strand 7 have shown relatively minimal effects on the GFP fluorescence.^21^ The ATCUN motif (Gly-Gly-His) was then inserted into the N-terminal region in a manner in which the histidine at the third position replaced histidine 148 near the phenolic ring of the chromophore (Fig. 1 and Table 1). To provide an α-NH_2_-terminal nitrogen for ATCUN in the circularly permuted GFP, a free N-terminus at the first glycine of the motif was precisely generated by intein-based protein cleavage.^22^ N-terminal sequencing of the intein-cleaved protein confirmed proper insertion of the ATCUN motif (Fig. S1†). For fabrication of the sensor protein, we employed superfolder GFP (OPT)^23^ for higher stability in the assay buffer (100 mM HEPES, pH 7.0, 300 mM NaCl) and enhanced folding in *E. coli* as well as in mammalian cells (data not shown).

**Table 1:** N-terminal sequences of GCS and control proteins. The ATCUN motif is shown in bold italic

Construct					+1	H148	–1				
cpOPT		NH_2_–	E	L	S	H	N	V	Y	I	T
GCS-1			NH_2_–	***G***	***G***	***H***	N	V	Y	I	T
(–1)GCS-1				NH_2_–	***G***	***G***	***H***	V	Y	I	T
GCS-2		NH_2_–	***G***	***G***	***H***	H	N	V	Y	I	T
G-GCS-2	NH_2_–	G	***G***	***G***	***H***	H	N	V	Y	I	T
GCS-2-G		NH_2_–	***G***	***G***	***H***	G	N	V	Y	I	T

**Fig. 2:**
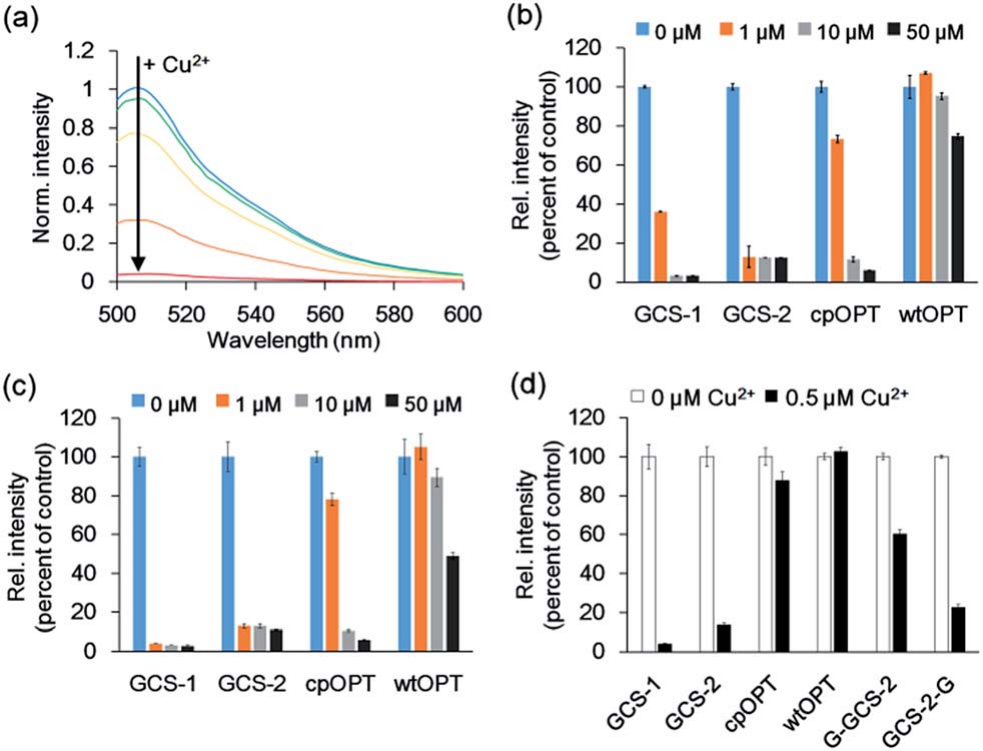
Fluorescence responses of GCS sensor proteins. (a) Fluorescence responses of 1 μM GCS-1 <5 min after the addition of 0, 0.4, 0.8, 1.6, and 3.2 μM Cu^2+^. (b) Fluorescence responses of 0.5 μM protein <5 min after the addition of 0, 1, 10, and 50 μM Cu^2+^. cpOPT: circularly permuted green fluorescent protein lacking the ATCUN motif; wtOPT: wild-type green fluorescent protein. (c) Fluorescence responses of 0.5 μM protein 1 h after the addition of 0, 1, 10, and 50 μM Cu^2+^. (d) Fluorescence responses of 0.1 μM protein 1 h after the addition of 0 and 0.5 μM Cu^2+^. The error bars correspond to the standard error of the mean of three independent measurements.

Fluorescence spectra of the circularly permuted OPT (cpOPT) with the ATCUN insert were acquired by exciting at 480 nm and collecting the emission signals at 500–600 nm. Upon the addition of Cu^2+^, the fluorescence intensity of the sensor protein clearly decreased in a concentration-dependent manner (Fig. 2a). The decrease in the fluorescence signal (at 510 nm) was up to <5% of the apo-protein fluorescence (95% quenched). Compared with wild-type OPT (wtOPT) and cpOPT, the ATCUN-fused cpOPT (GCS-1) exhibited a considerably higher degree of quenching over a wide range of Cu^2+^ concentrations (Fig. 2b). Although cpOPT displayed noticeable fluorescence quenching at high Cu^2+^ concentrations (>10 μM), introduction of the ATCUN motif increased the Cu^2+^ sensitivity of the protein. In addition, longer incubation of the ATCUN-fused cpOPT at low Cu^2+^ concentrations further enhanced fluorescence quenching, which was likely due to the rather slow Cu^2+^ binding by ATCUN in cpOPT (Fig. 2c). We named the sensor protein as genetically-encoded copper(ii) sensor (GCS)-1 (see Table 1 for a summary of N-terminal sequences in the GCS proteins).

To further optimize the Cu^2+^ responses, the location of the ATCUN motif in cpOPT was varied around H148 in GCS-1. ATCUN insertion at the –1 position of H148 ((–1)GCS-1, Table 1) resulted in a sensor protein with Cu^2+^ binding responses similar to those of GCS-1 but a poor protein yield (data not shown). In contrast, cpOPT with the ATCUN motif at the +1 position of H148 (GCS-2) showed rapidly saturated fluorescence quenching even at low concentrations of Cu^2+^ (Fig. 2b and c), possibly indicating a higher Cu^2+^ binding affinity. Although the degree of quenching was slightly lower for GCS-2 (<15% of apo-protein fluorescence, >85% quenched), the binding response of GCS-2 was significantly faster (within 10 s) than that of GCS-1 (>20 min, Fig. S2†). The improved response rate of GCS-2 might be due to an increased flexibility of the ATCUN motif in the protein. Unlike GCS-1, the ATCUN motif in GCS-2 does not include H148 which is known to interact with neighboring β-strands.23*a* Thereby the ATCUN tripeptide of GCS-2 can be more open for Cu^2+^ binding, exhibiting a faster association. On the other hand, H148 is also in a hydrogen-bonding network that stabilizes the chromophore.^24^ Conformational changes of H148 and subsequent effects on the chromophore by Cu^2+^ binding might be less significant in GCS-2 than in GCS-1. The absorbance spectrum of GCS-1 was altered more strongly by Cu^2+^ binding than that of GCS-2 (Fig. S3†), possibly indicating stronger influences on the chromophore and thereby a higher degree of quenching response in GCS-1.

To demonstrate that the ATCUN motif is indeed the major Cu^2+^ binding site in GCS-2, we constructed two GCS-2 variants, G-GCS-2 and GCS-2-G (Table 1). The ATCUN motif in G-GCS-2 was disrupted by adding a glycine residue to the N-terminus of GCS-2. Upon the addition of Cu^2+^, G-GCS-2 showed a severely diminished quenching response (Fig. 2d). On the other hand, the fluorescence intensity of GCS-2-G, in which the ATCUN motif is intact but H148 was mutated to glycine, was still reduced to <30% of the apo-protein fluorescence (>70% quenched) under the same conditions, supporting that the ATCUN motif is primarily responsible for Cu^2+^ binding (Fig. 2d). In addition, titration with Cu^2+^ revealed a 1 : 1 binding stoichiometry between GCS-2 and Cu^2+^ (Fig. S4†), suggesting the presence of a single dominant metal binding site (such as the ATCUN tripeptide) in GCS-2. However, it should be noted that H148 of GCS-2 also plays a partial role in Cu^2+^ binding and the resulting fluorescence quenching since the Cu^2+^-mediated response of GCS-2-G was smaller than that of GCS-2.

**Fig. 3:**
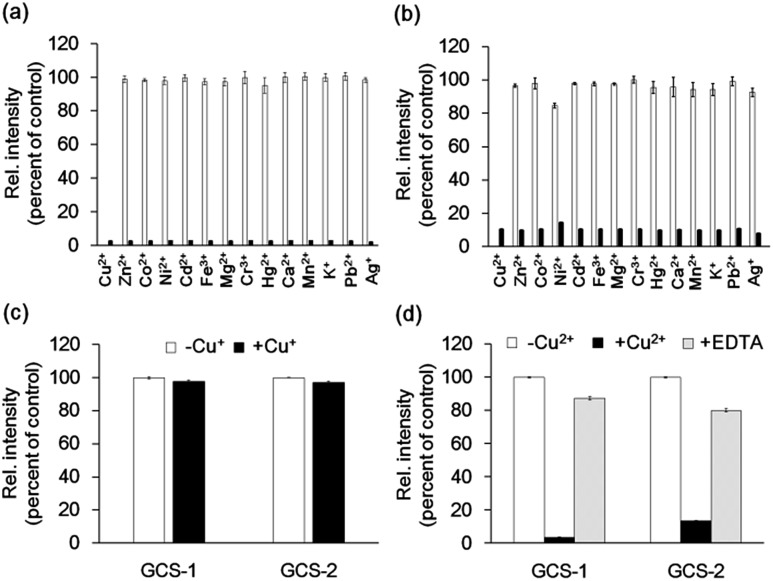
Cu^2+^ selectivity and reversibility of GCS proteins. (a and b) Fluorescence responses of 1 μM GCS-1 (a) and GCS-2 (b) to various metal ions. White bars represent fluorescence responses with 5 μM of the respective metal ions. Black bars represent fluorescence responses after the addition of 5 μM Cu^2+^. (c) Fluorescence responses of 1 μM GCS protein with 5 μM Cu^+^ including 250 μM of l-ascorbic acid to prevent oxidation of Cu^+^ to Cu^2+^. Fluorescence spectra were collected within 5 min after Cu^+^ addition. (d) Fluorescence responses of 1 μM GCS proteins with 5 μM Cu^2+^ followed by the addition of 50 μM EDTA. All responses were normalized against the fluorescence intensity of 1 μM GCS protein lacking a metal ion. The error bars correspond to the standard error of the mean of three independent measurements.

The metal selectivity of both GCS proteins was subsequently investigated by monitoring changes in fluorescence intensity in the GCS proteins when other biologically relevant metal ions (Cr^3+^, Mn^2+^, Fe^3+^, Co^2+^, Ni^2+^, Zn^2+^, Ag^+^, Cd^2+^, Hg^2+^, Pb^2+^, K^+^, Ca^2+^, and Mg^2+^) were present. Most of these metal ions had no effect on the fluorescence quenching by Cu^2+^ (Fig. 3a and b). Although Ni^2+^ caused a small fluorescence change for GCS-2, subsequent Cu^2+^ addition readily induced fluorescence quenching (Fig. 3b). The ATCUN motif in our GCS sensor proteins likely binds to Ni^2+^ more weakly than to Cu^2+^, which is similar to the ATCUN motif in HSA.^20^ The effects of Cu^+^ were examined in a buffer containing a 50-fold molar excess of l-ascorbic acid. l-ascorbic acid had no influence on the fluorescence intensities of the GCS proteins (Fig. S5†) or Cu^2+^ binding of the ATCUN motifs.^25^ Cu^+^ addition showed no change in fluorescence for both GCS proteins (Fig. 3c). The reversibility of the fluorescence quenching of the sensor protein was also tested, and fluorescence signals from the Cu^2+^-bound GCS proteins were recovered upon addition of metal-chelating ethylenediaminetetraacetic acid (EDTA, Fig. 3d).

**Fig. 4:**
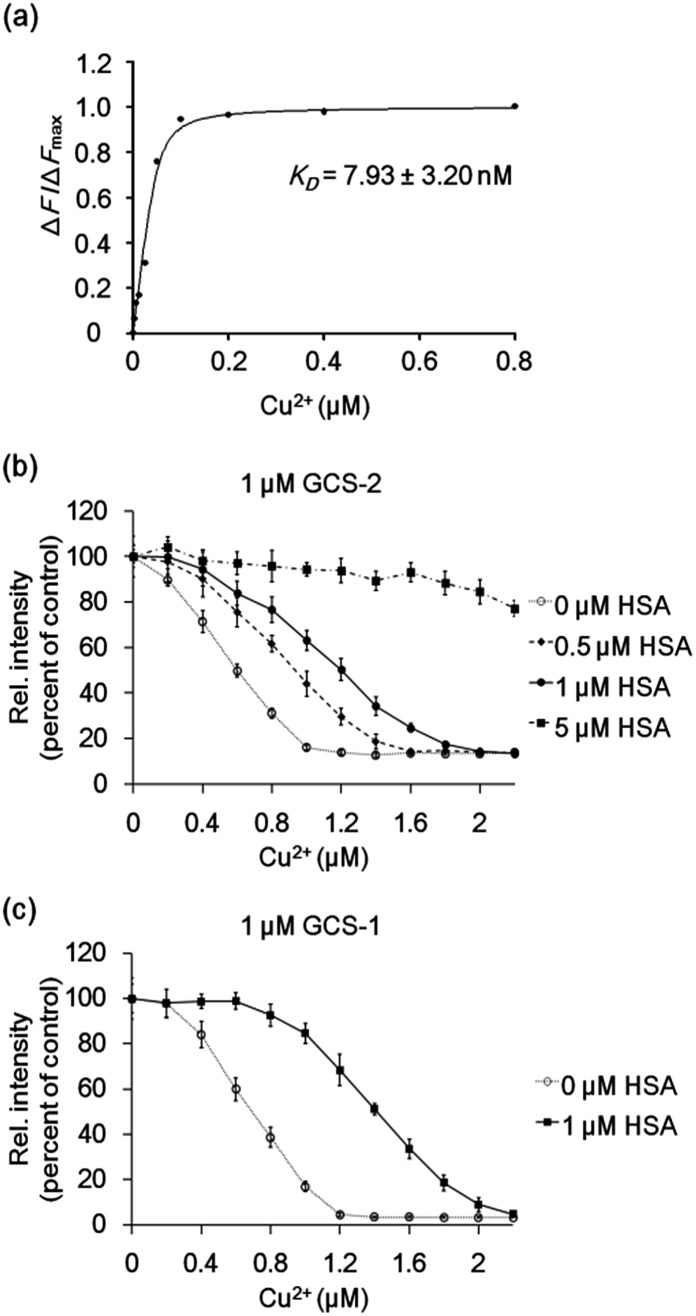
(a) Representative plot of Δ*F*/Δ*F*_max_ of 50 nM GCS-2 with various Cu^2+^ concentrations. The binding constant *K*_D_ was derived from three independent experiments. Δ*F*: change in measured fluorescence, Δ*F*_max_: maximum fluorescence change. (b) GCS-2 fluorescence changes with varying concentration of Cu^2+^ in the absence and presence of 0.5, 1, and 5 μM HSA. (c) GCS-1 fluorescence changes with varying concentration of Cu^2+^ in the absence and presence of 1 μM HSA. The error bars correspond to the standard error of the mean of three independent measurements.

The Cu^2+^-binding affinities of the GCS proteins were determined by measuring fluorescence changes upon titration with copper.15*b* All assays were performed at relatively high buffer and salt concentrations (100 mM HEPES, pH 7.0, 300 mM NaCl), which might reflect various extracellular media.^26^ The dissociation constant for GCS-2 was calculated to be 7.93 ± 3.20 nM (Fig. 4a), which is the strongest Cu^2+^ binding among the currently available protein-based Cu^2+^ sensors (Table S1†). The use of a natural copper(ii)-specific tripeptide provides a strong chelating strategy for robust Cu^2+^ capture near the GFP chromophore, presenting high sensitivity for the metal ion while not compromising the metal selectivity of the GCS proteins. GCS-1 was found to bind Cu^2+^ more weakly (94.04 ± 19.33 nM) as discussed above (Fig. S6†).

To test whether the binding affinity of the GCS proteins for Cu^2+^ is high enough to monitor the metal ion even in the presence of other copper-binding molecules, the GCS responses to Cu^2+^ were measured in the presence of various amounts of HSA. GCS-2 showed Cu^2+^ binding properties comparable to HSA, where >40% of the added Cu^2+^ was bound to GCS-2 in the presence of equimolar HSA (Fig. 4b). Considering that the reported dissociation constants for HSA are in the picomolar range,^13^ Cu^2+^ binding to GCS-2 should be weaker by at least three orders of magnitude. This discrepancy is likely due to the different experimental methods used for *K*_D_ measurements, components/concentration of buffers, pH, or ionic strengths.^27^ Nevertheless, under our conditions, an optimized ATCUN tripeptide in GCS-2 showed nearly as strong binding as the ATCUN motif in HSA. In contrast, the fluorescence intensity of GCS-1 began to decrease only after the concentration of Cu^2+^ was high enough to fill in most of the ATCUN sites in HSA (Fig. 4c). Therefore, GCS-1 was not able to effectively compete with HSA. We also performed competing experiments with an amyloid beta peptide (Aβ40), another copper-binding molecule (*K*_D_ 0.47–30 μM) which plays a key role in the pathogenesis of Alzheimer's disease.^28^ Aβ40, which has a rather weak Cu^2+^-binding ability, was outcompeted by both GCS proteins (Fig. S7†). Taken together, our data show that GCS-2 (*K*_D_ ∼ 8 nM) is a highly sensitive reporter that can efficiently compete with other high-affinity copper-binding molecules in extracellular media. In comparison, GCS-1 (*K*_D_ ∼ 94 nM) might be useful for applications in which low-affinity reporter proteins are required.

**Fig. 5:**
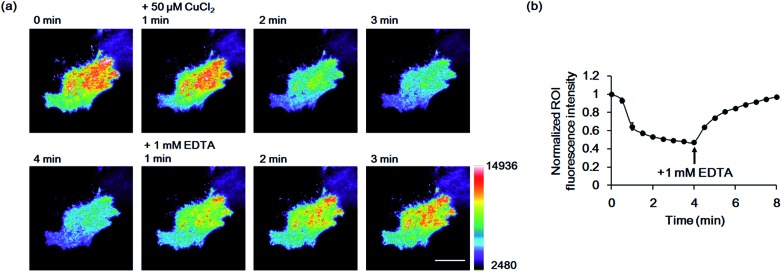
Imaging of Cu^2+^ on live HeLa cell surfaces by GCS-2. (a) Pseudocolored images from 1 min prior to Cu^2+^ addition to 1, 2, 3 and 4 min after the addition of 50 μM Cu^2+^, and 1, 2 and 3 min after the addition of 1 mM EDTA. Images were collected using total internal reflection fluorescence (TIRF) microscopy. The color bar represents the fluorescence intensity in the GFP channel (excitation at 488 nm). Scale bar: 20 μm. (b) Plot of the average fluorescence response of 13 cells *vs.* time after 50 μM Cu^2+^ addition. All fluorescence intensity values were subtracted by background signals and normalized by the fluorescence before Cu^2+^ addition. The time of 1 mM EDTA addition is shown. The error bars correspond to the standard error of the mean value of 13 individual cells.

Finally, we used the GCS proteins to detect dynamic Cu^2+^ fluctuations on the surfaces of live mammalian cells. A signal peptide for the secretory pathway and the transmembrane domain of the platelet-derived growth factor receptor were fused to GCS-2 for display on the extracellular plasma membrane. The signal peptide is cleaved during sensor protein synthesis in the endoplasmic reticulum, generating the ATCUN motif precisely at the N-terminus for effective Cu^2+^ binding. HeLa cells expressing the GCS-2 construct were monitored with a total internal reflection fluorescence (TIRF) microscope for selective visualization of the cell surface fluorescence. Upon Cu^2+^ addition (50 μM), the GCS-2 fluorescence intensity on the cell surface was rapidly decreased within ∼1.5 min, and it was saturated to 47% (average from 13 cells in the same well, 53% quenched) of the initial signal after 3 min (Fig. 5). EDTA was then added to the cells, and the GCS-2 fluorescence intensity was recovered to 97% within 4 min, which is in agreement with the *in vitro* results. GCS-1 that was expressed on HeLa cell surfaces provided fluorescence changes similar to those of GCS-2 against extracellular Cu^2+^ (data not shown).

**Fig. 6:**
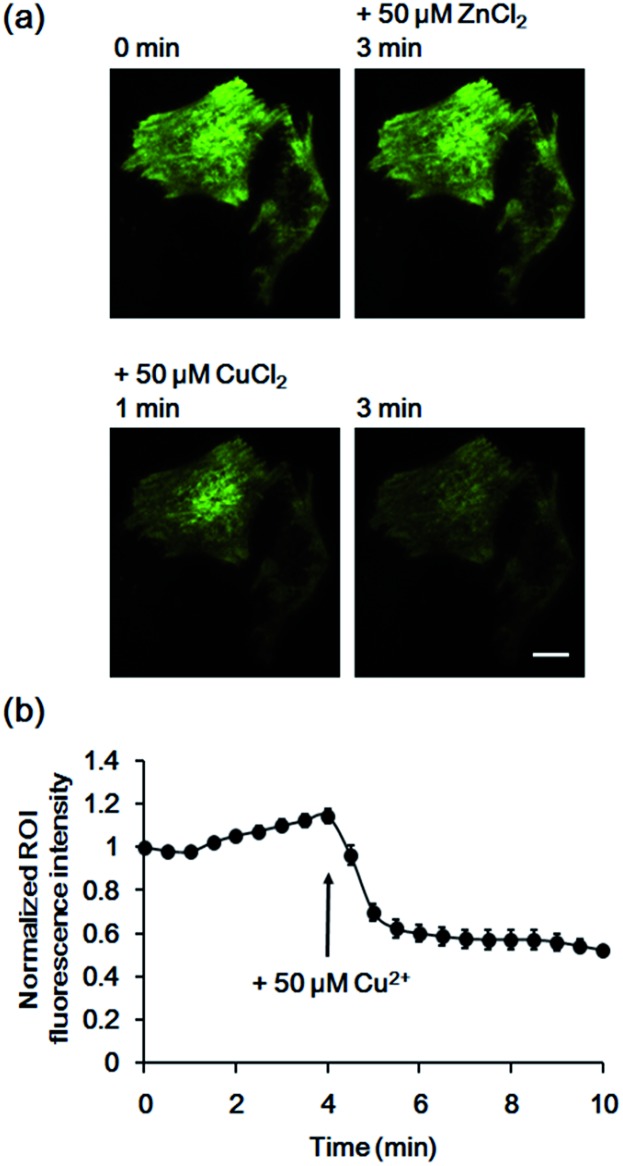
Fluorescence response of GCS-2 to 50 μM Zn^2+^ on live HeLa cell surfaces followed by addition of 50 μM Cu^2+^. (a) Images of 1 min prior to Zn^2+^ addition, 3 min after the addition of Zn^2+^, and 1 and 3 min after the addition of Cu^2+^. Scale bar: 10 μm. (b) Plot of the average response of 13 cells *vs.* time after 50 μM Zn^2+^ addition. All fluorescence intensity values were subtracted by background signals and normalized by the fluorescence before Zn^2+^ addition. The time of 50 μM Cu^2+^ addition is shown. The error bars correspond to the standard error of the mean value of 13 individual cells.

**Fig. 7:**
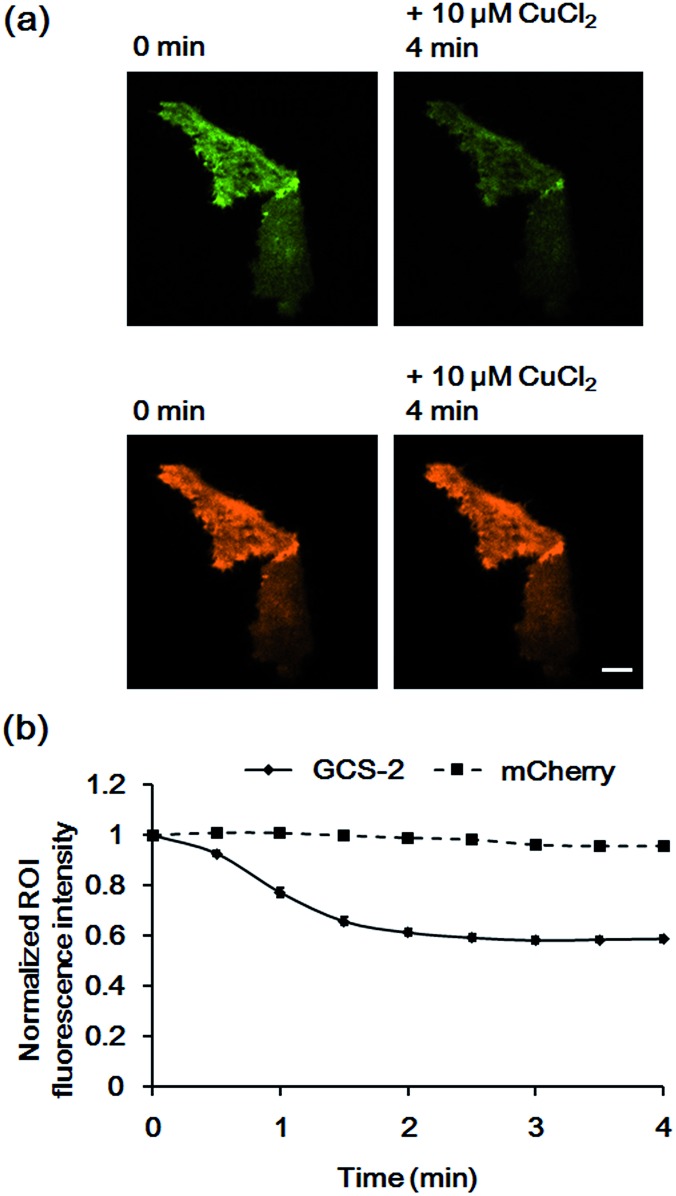
Fluorescence responses of GCS-2 and mCherry co-expressed in HeLa cells to 10 μM Cu^2+^. (a) Images of GCS-2 (top) and mCherry (bottom) of 1 min prior to Cu^2+^ addition and 4 min after the addition of Cu^2+^. Scale bar: 10 μm (b) plots of the average fluorescence responses of GCS-2 and mCherry of 8 cells *vs.* time after 10 μM Cu^2+^ addition. All fluorescence intensity values were subtracted by background signals and normalized by the fluorescence before Cu^2+^ addition. The error bars correspond to the standard error of the mean value of eight individual cells.

To confirm that the decrease in fluorescence of GCS-2 was specific for Cu^2+^ binding, Zn^2+^, another crucial metal ion in the brain, was added to the cells (50 μM) before Cu^2+^ treatment. Interestingly, the fluorescence intensity of several cells of the 13 monitored cells was slightly increased (<115%) by Zn^2+^ (Fig. 6). This could result from structural stabilization of the β-barrel of the GCS-2 protein under relatively high zinc concentrations as recently reported for a red fluorescent mKate2 mutant.11*a* Zinc(ii)-mediated changes in protein distribution in the plasma membrane might also affect the fluorescence intensity of GCS-2 as observed using TIRF imaging. Nevertheless, all cells exhibited rapid fluorescence quenching upon Cu^2+^ addition (Fig. 6). The copper(ii)-specific fluorescence decrease for GCS-2 was further confirmed by its co-expression with mCherry, a red fluorescent protein, on the cell surfaces. Addition of 10 μM CuCl_2_ decreased the fluorescence intensity of GCS-2 but not that of mCherry, which again shows the applicability of GCS-2 for specific Cu^2+^ imaging on the surface of live cells (Fig. 7). Moreover, the fluorescence signals of cpOPT proteins on cell surfaces were not altered by 10 μM Cu^2+^ (Fig. S8†).

## Conclusions

The GCS proteins demonstrate that the natural and small Cu^2+^ binding ATCUN motif can be successfully utilized as a strong and specific copper chelating site for genetically encoded Cu^2+^ reporters. The tight Cu^2+^ binding of GCS-2 allows for reliable Cu^2+^ monitoring in the presence of HSA, one of the major Cu^2+^ binding biomolecules in extracellular environments. The sensor proteins also demonstrate high selectivity for Cu^2+^ compared with other biologically relevant metal ions. Imaging Cu^2+^ on the surface of live cells with GCS-2 showed a quick and reversible response, which demonstrates the possibility of monitoring oscillating Cu^2+^ levels in living systems. The copper-chelating strategy described herein has multiple advantages. Taken from one of the major players in extracellular copper trafficking, the ATCUN motif may enable non-invasive detection of dynamic changes in copper concentration in biological systems. The motif is composed of only three amino acids, creating minimal disruption in the fluorescent protein’s structure. To expand the applicability of GCS sensors to intracellular Cu^2+^ imaging, strategies to generate ATCUN motifs and protect these motifs from various post-translational modifications^29^ inside cells should be developed. Nonetheless, we envision that GCS proteins can be employed to monitor Cu^2+^ release on neuronal cells. Specifically, copper release in the synaptic cleft which is induced by chemical or biological cues could be detected by GCS proteins expressed on pre- or postsynaptic cell surfaces. Further studies will be aimed at improving the membrane targeting efficiency and applications of red fluorescent proteins that enable Cu^2+^ imaging in live animals.

## Supplementary Material

Supplementary information
