# Supplementary material for: A novel copper-chelating strategy for fluorescent proteins to image dynamic copper fluctuations on live cell surfaces
Source: Chem Sci. 2014 Nov 19;6(2):1301–7. doi: 10.1039/c4sc03027c (PMC5811147; doi:10.1039/c4sc03027c)
Supplement: Supplementary file 1 [file SC-006-C4SC03027C-s001.pdf]

Electronic Supplementary Material (ESI) for Chemical Science.
This journal is © The Royal Society of Chemistry 2014

Electronic Supplementary Information

A novel copper-chelating strategy for fluorescent proteins to image dynamic copper fluctuations on live cell surfaces

Yoon-Aa Choi, Joo Oak Keem, Cha Yeon Kim, Hye Ryeon Yoon, Won Do Heo, Bong Hyun Chung,* and Yongwon Jung*

	page
Supporting Figures	S2-S15
Supporting Table	S16
Experimental Methods	S17-S28

Supporting Figure

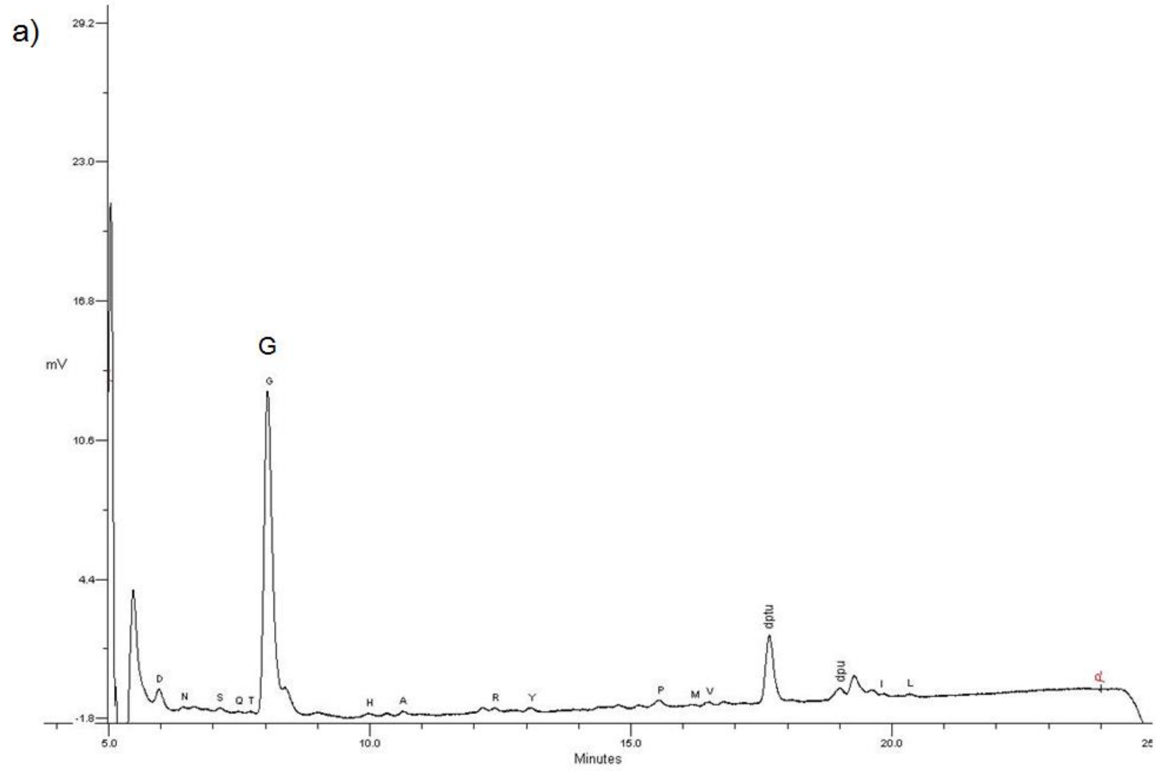

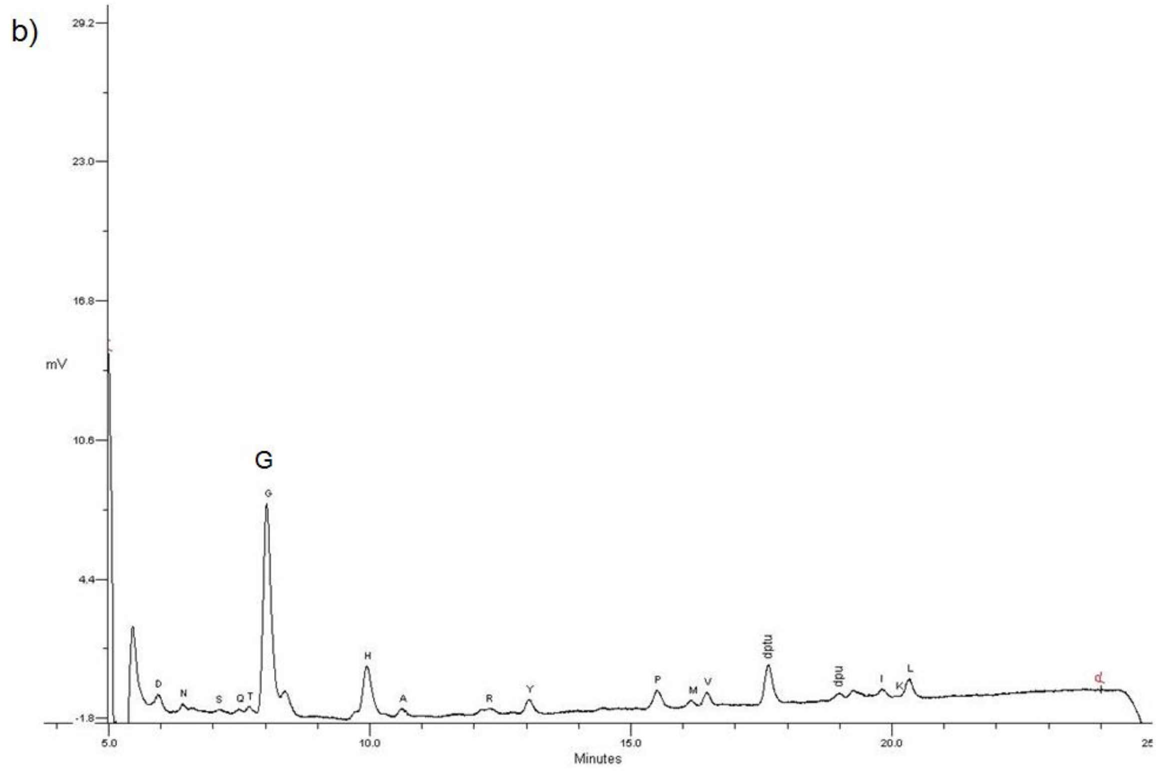

c)

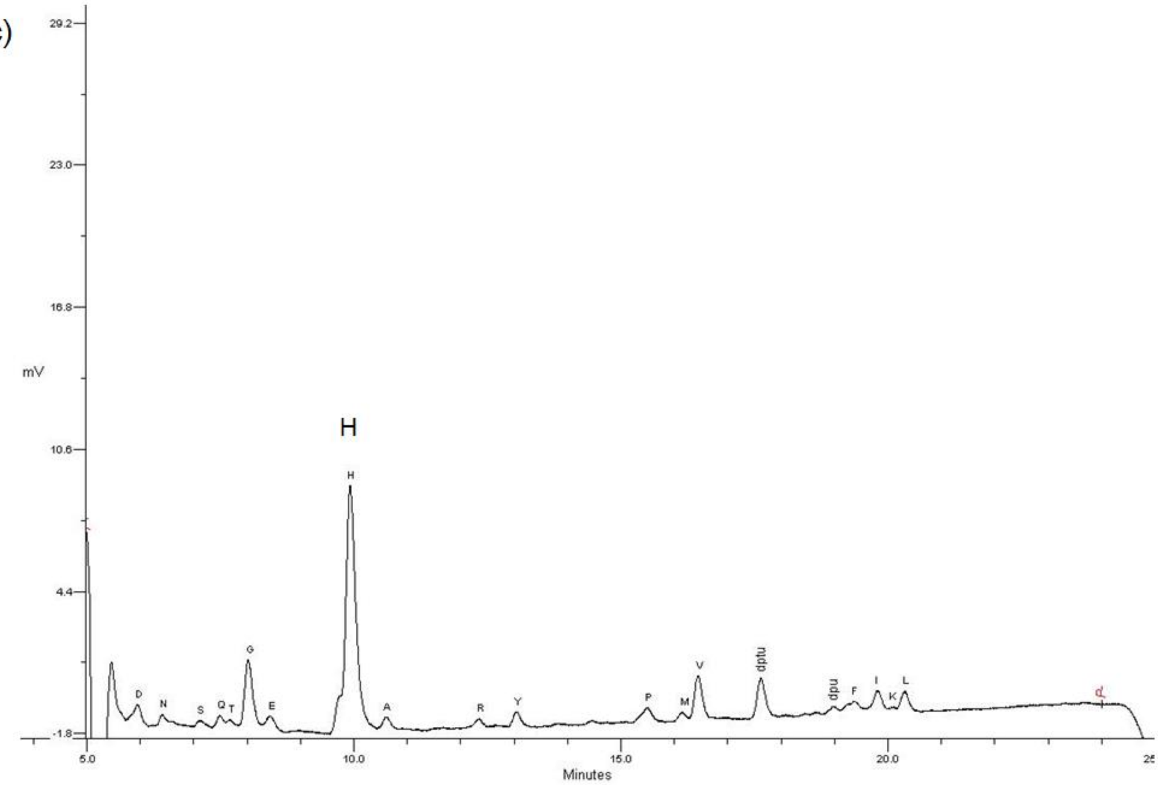
29.2
23.0
16.8
mV
10.6
4.4
-1.8
H
D
N
S
Q
T
G
E
A
R
Y
P
M
V
dptu
dpu
F
I
K
L
5.0
10.0
15.0
20.0
Minutes

d)

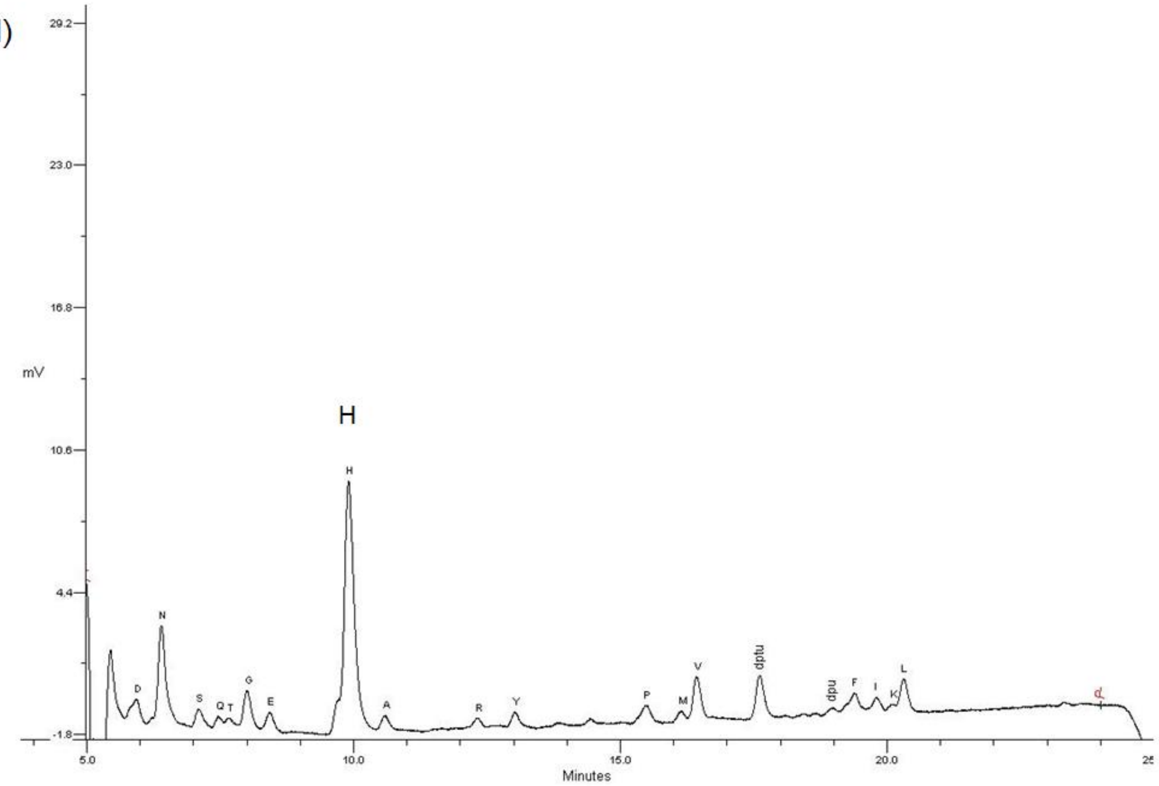
29.2
23.0
16.8
mV
10.6
4.4
-1.8
H
D
N
S
Q
T
G
E
A
R
Y
P
M
V
dptu
dpu
F
I
K
L
5.0
10.0
15.0
20.0
Minutes

e)

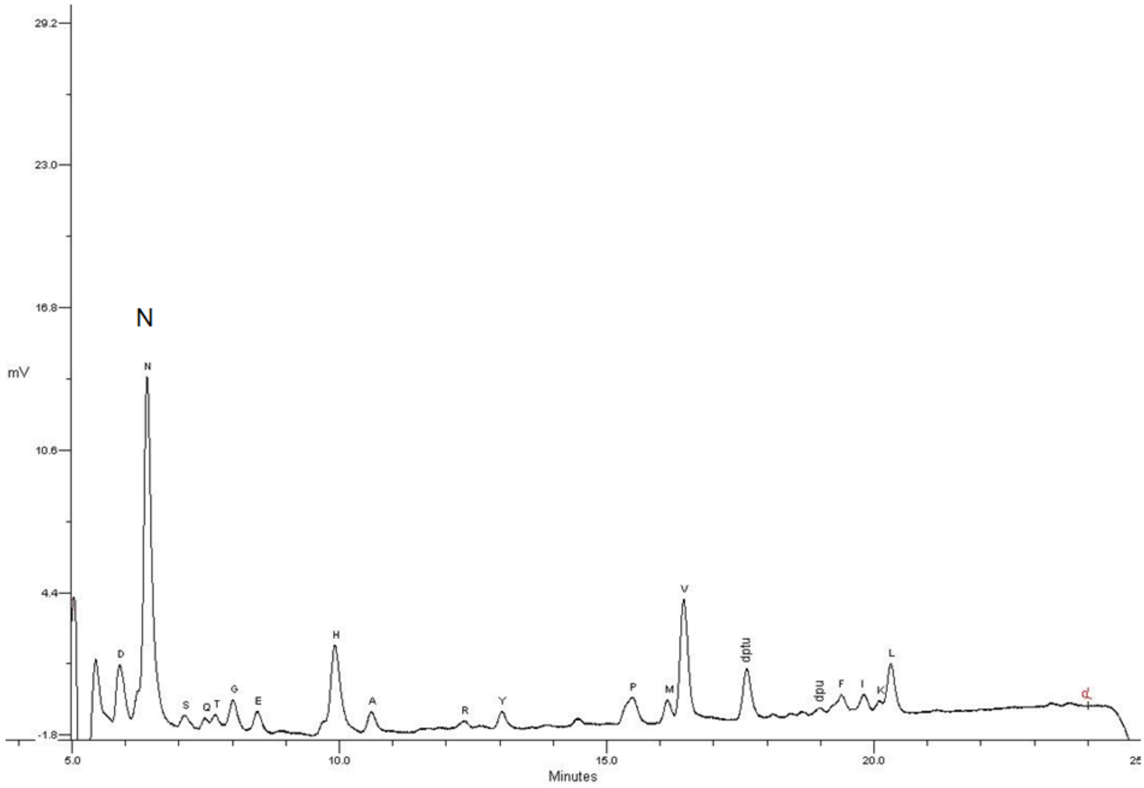

29.2
23.0
16.8
10.6
4.4
-1.8
mV
N
D
S
Q
T
G
E
H
A
R
Y
P
M
V
dptu
dpu
F
I
K
L
5.0
10.0
15.0
20.0
Minutes

f)

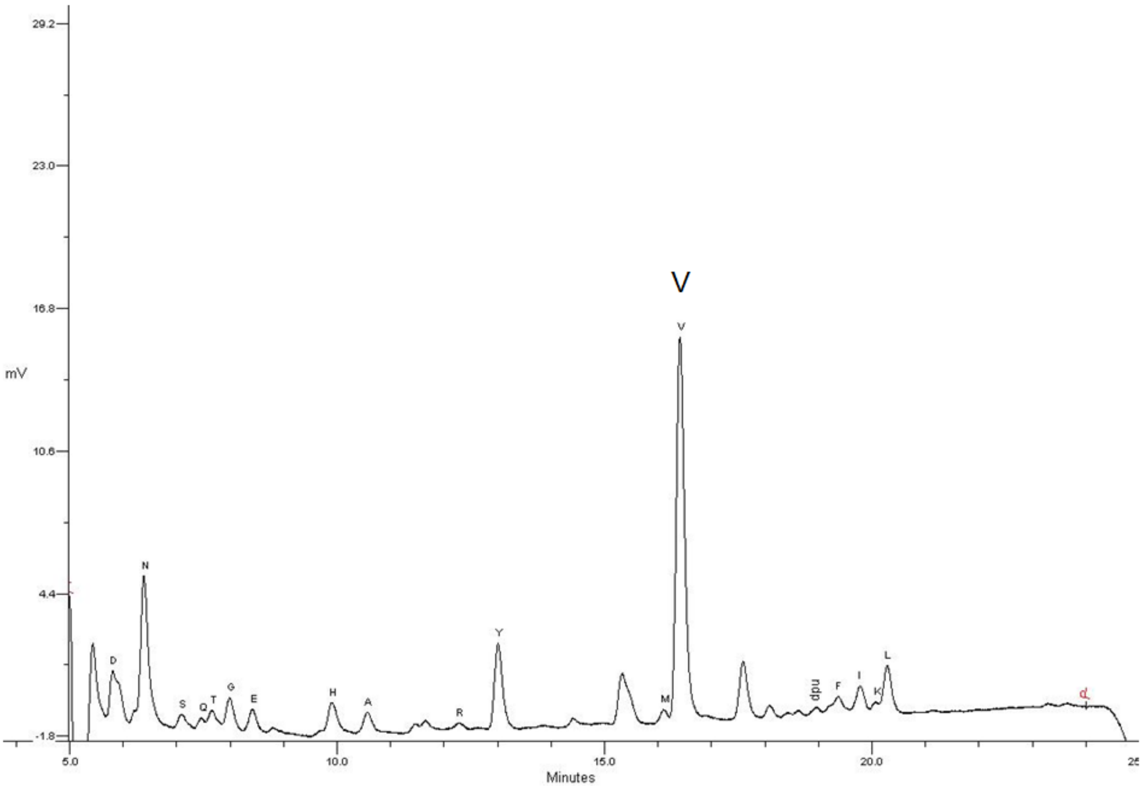

29.2
23.0
16.8
10.6
4.4
-1.8
mV
V
N
D
S
Q
T
G
E
H
A
R
Y
M
dpu
F
I
K
L
5.0
10.0
15.0
20.0
Minutes

Fig. S1 (previous pages) N-terminal sequencing profiles of ATCUN-fused circularly permuted EGFP (N-terminal sequence: **GGH**HNVYI, the ATCUN motif is shown in bold), which is purified by intein cleavage. a) residue #1, b) residue #2, c) residue #3, d) residue #4, e) residue #5, and f) residue #6. The main peak of each chromatogram was labeled with the identified residue.

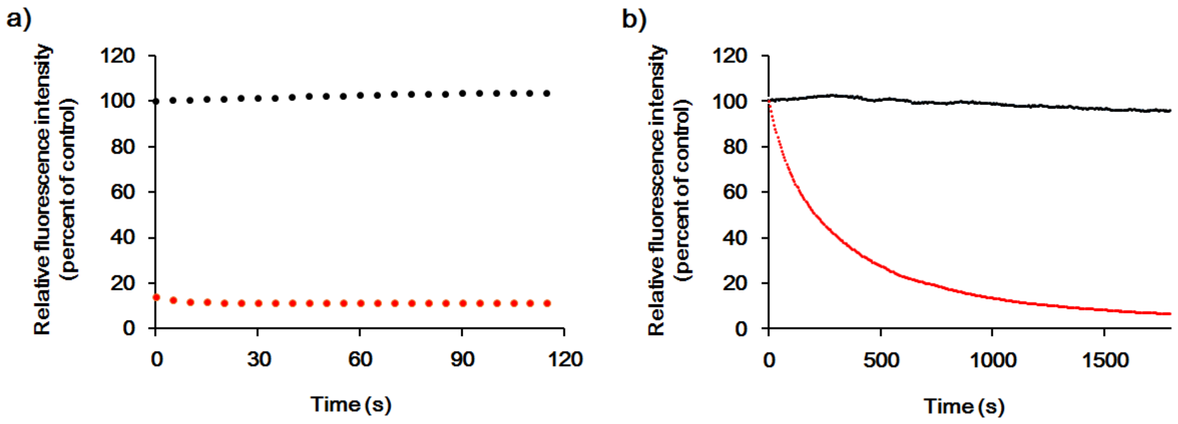

Fig. S2 Time-dependent fluorescence responses of a) GCS-2 and b) GCS-1 upon Cu^{2+} addition. GCS proteins (0.5 μM) were treated with 1 μM of Cu^{2+}, and the fluorescence intensity was measured every 5 s. Black dots represent the fluorescence intensities of GCS proteins without Cu^{2+}. Red dots represent the fluorescence intensities of GCS proteins with Cu^{2+}.

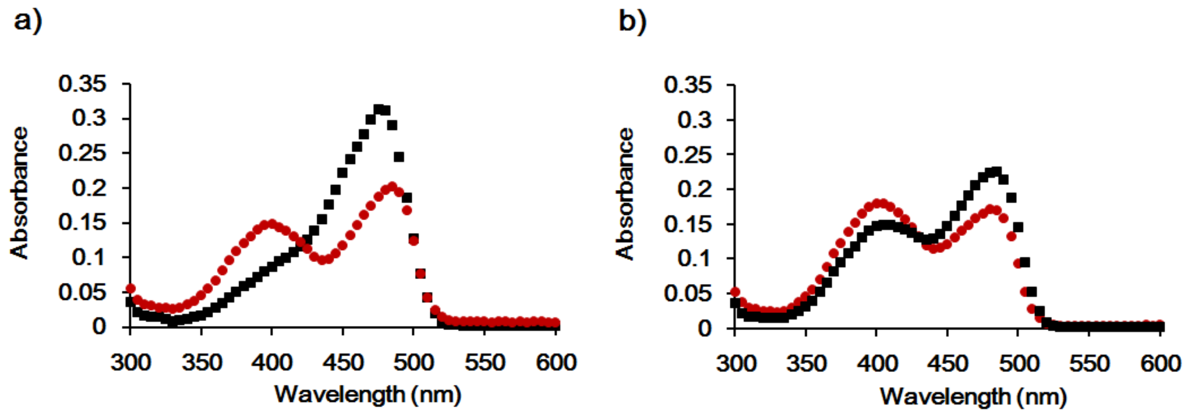

Fig. S3 UV-VIS spectra of a) GCS-1 and b) GCS-2 with (red dots) or without (black dots) Cu^{2+}. 10 μM proteins were mixed with 20 μM Cu^{2+}.

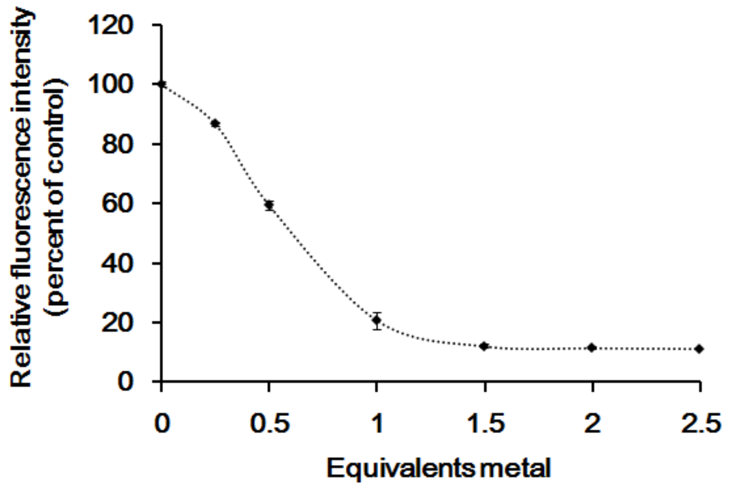

Fig. S4 Fluorescence response of GCS-2 upon the addition of various equivalents of Cu^{2+}. GCS-2 (10 μM) was titrated with 0, 5, 10, 15, 20, and 25 μM Cu^{2+}. The error bars correspond to the standard error of the mean of three independent measurements.

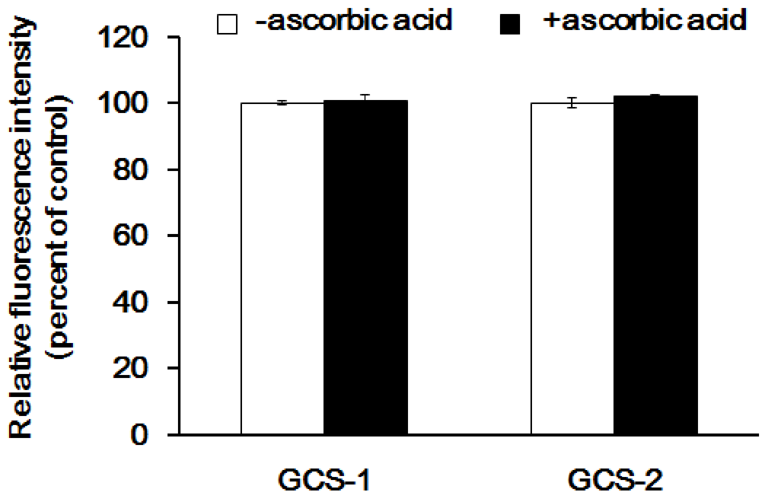

Fig. S5 Fluorescence responses of GCS proteins to L-ascorbic acid. Relative fluorescent intensities of 1 μM GCS proteins with (black bars) and without (white bars) 250 μM L-ascorbic acid are shown. Fluorescence spectra were collected within 5 min after L-ascorbic acid addition. The error bars correspond to the standard error of the mean of three independent measurements.

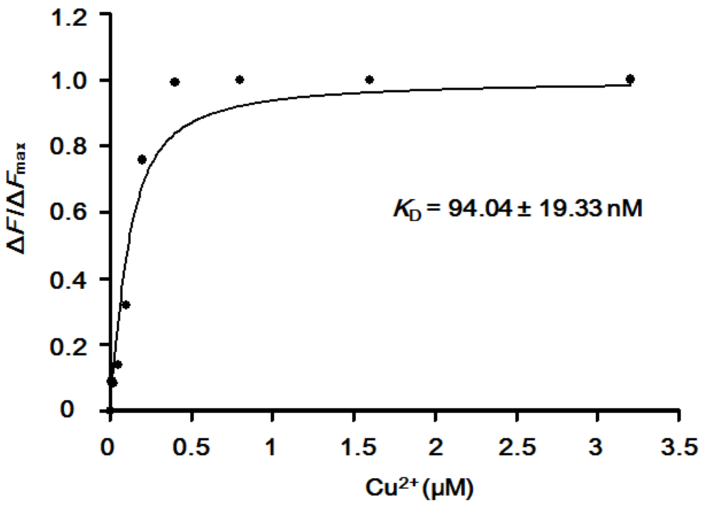

Fig. S6 Representative plot of $\Delta F/\Delta F_{max}$ of 100 nM GCS-1 against various Cu^{2+} concentrations. The binding constant K_D was derived from three independent experiments. ΔF: change in measured fluorescence, ΔF_{max}: maximum fluorescence change.

a)

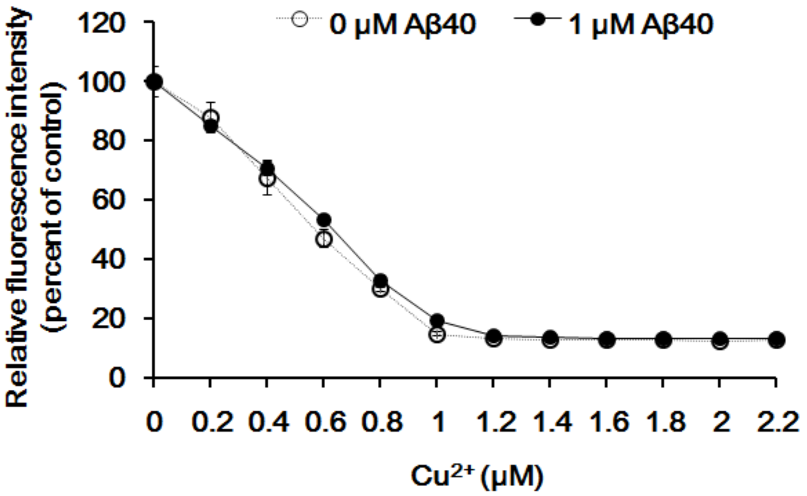

b)

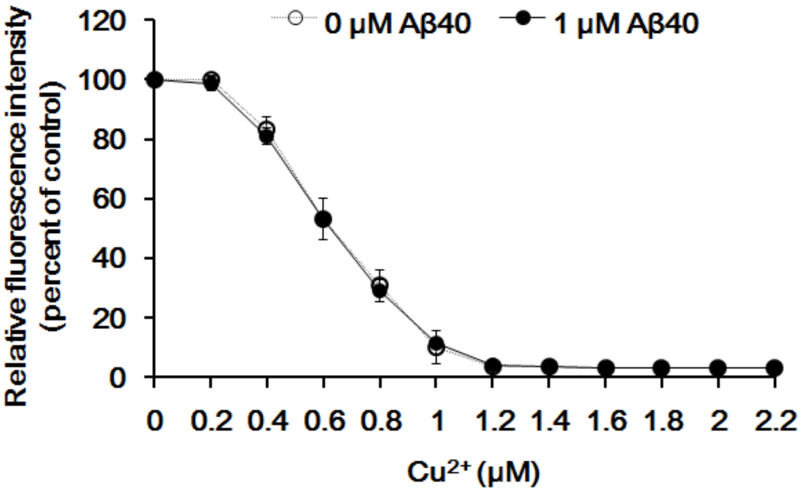

Fig. S7 Competition between GCS proteins and Aβ40 for Cu^{2+} binding. a) Fluorescence changes of (a) GCS-2 and (b) GCS-1 by varying concentrations of Cu^{2+} in the absence (blank circles) and presence (black circles) of 1 μM Aβ40. The error bars correspond to the standard error of the mean of two independent measurements.

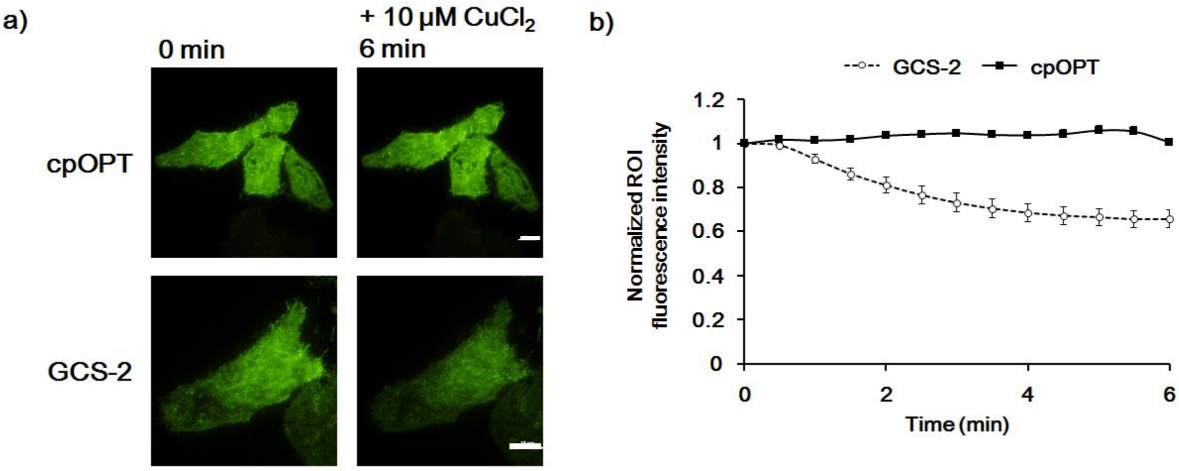

Fig. S8 Fluorescence responses of cpOPT and GCS-2 on HeLa cell surfaces to 10 μM Cu^{2+}. a) Fluorescence Images of cells with cpOPT (top) and GCS-2 (bottom). Images were obtained at 1 min prior to Cu^{2+} addition and 6 min after Cu^{2+}addition of. Scale bars (white rectangles) correspond to 10 μm. b) Plots of the average fluorescence responses of GCS-2 and cpOPT vs. time after 10 μM Cu^{2+} addition. All fluorescence intensity values were subtracted by background signals and normalized by the fluorescence before Cu^{2+} addition. The error bars correspond to the standard error of the mean of 18 (GCS-2) and 17 (cpOPT) individual cells.

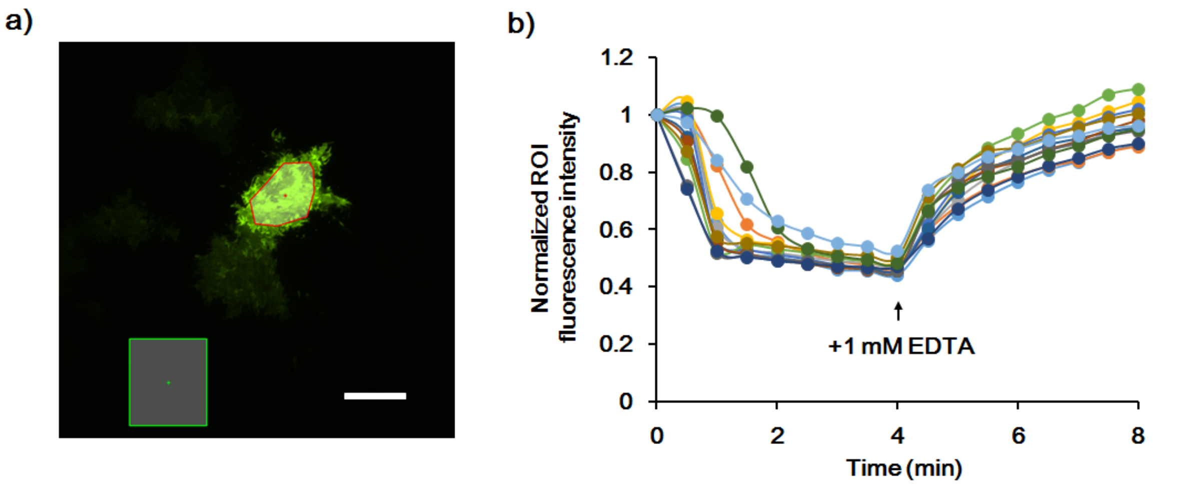

Fig. S9 Quantitative analysis of live-cell fluorescence images of GCS-2. a) Representative regions of interest (ROIs) drawn for cellular response (red polygon) and the background (green rectangle). Scale bar (white rectangle) corresponds to 10 μm. b) Plots of fluorescence responses of 13 cells vs. time after 50 μM Cu^{2+} addition. All fluorescence intensity values were subtracted by background signals and normalized by the fluorescence before Cu^{2+} addition. The time of 1 mM EDTA addition is shown.

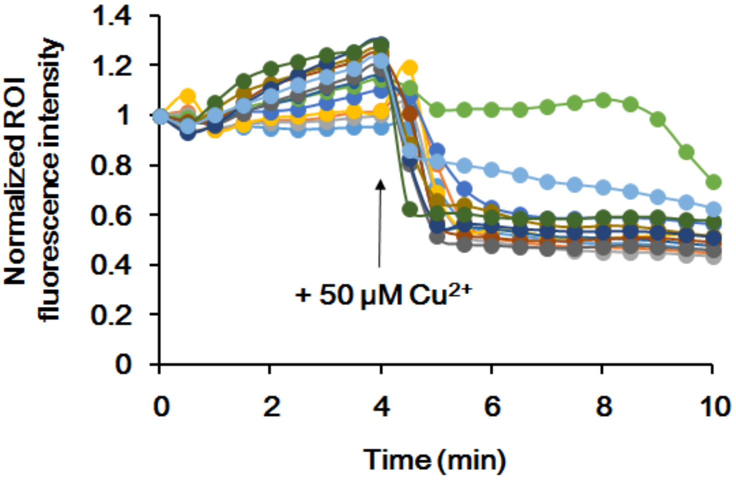

Fig. S10 Plots of Fluorescence responses of GCS-2 to 50 μM Zn^{2+} and subsequent 50 μM Cu^{2+} on live HeLa cell surfaces. Fluorescent signal changes of 13 cells vs. time after 50 μM Zn^{2+} and 50 μM Cu^{2+} additions are plotted. All fluorescence intensity values were subtracted by background signals and normalized by the fluorescence before Zn^{2+} addition. The time of 50 μM Cu^{2+} addition is shown.

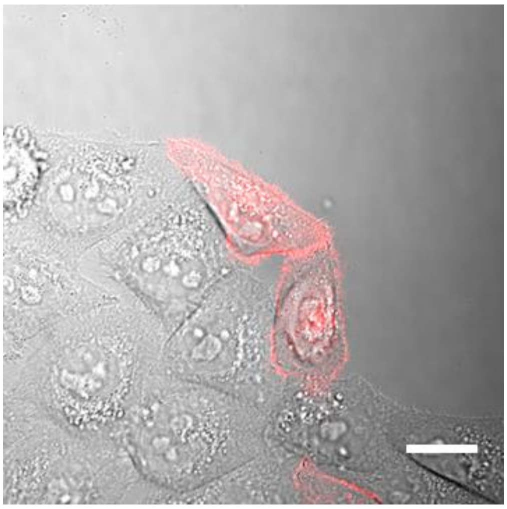

Fig. S11 Differential interference contrast (DIC) image of observed cells overlaid with a fluorescence image of mCherry. The image was acquired by confocal microscopy after TIRF imaging Scale bar (white rectangle) corresponds to 10 μm.

```
>GCS-1
GGHNVYITADKQKNGIKANFTVRHNVEDGSVQLADHYQQNTPIGDGPVLLP
DNHYLSTQTVLSKDPNEKRDHMVLLEFVTAAGITHGMDELYKGGTGGSMSK
GEELFTGVVPILVELDGDVNGHKFSVRGEGEGDATIGKLTLKFICTTGKLPVP
WPTLVTTLTYGVQCFSRYPDHMKRHDFFKSAMPEGYVQERTISFKDDGKY
KTRAVVKFEGDTLVNRIELKGTDFKEDGNILGHKLEYN
```

```
>GCS-2
GGHHNVYITADKQKNGIKANFTVRHNVEDGSVQLADHYQQNTPIGDGPVLL
PDNHYLSTQTVLSKDPNEKRDHMVLLEFVTAAGITHGMDELYKGGTGGSM
SKGEELFTGVVPILVELDGDVNGHKFSVRGEGEGDATIGKLTLKFICTTGKLP
VPWPTLVTTLTYGVQCFSRYPDHMKRHDFFKSAMPEGYVQERTISFKDDG
KYKTRAVVKFEGDTLVNRIELKGTDFKEDGNILGHKLEYN
```

Fig. S12 Sequence information of GCS proteins.

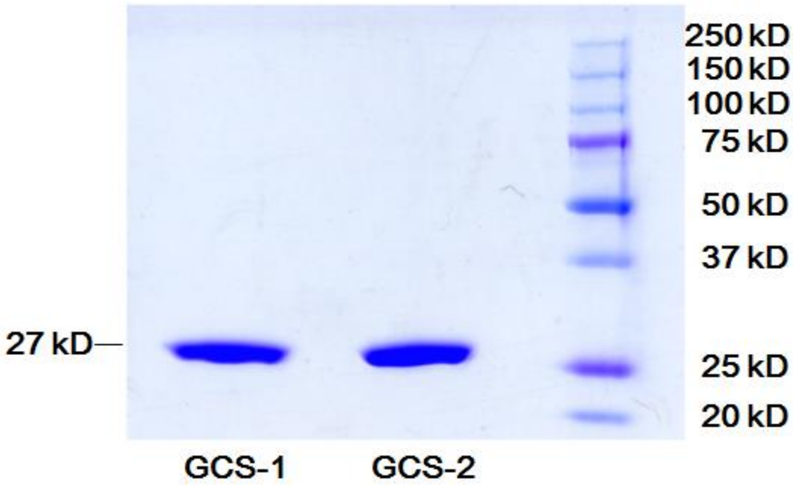

Fig. S13 SDS-PAGE analysis of the purified GCS proteins.

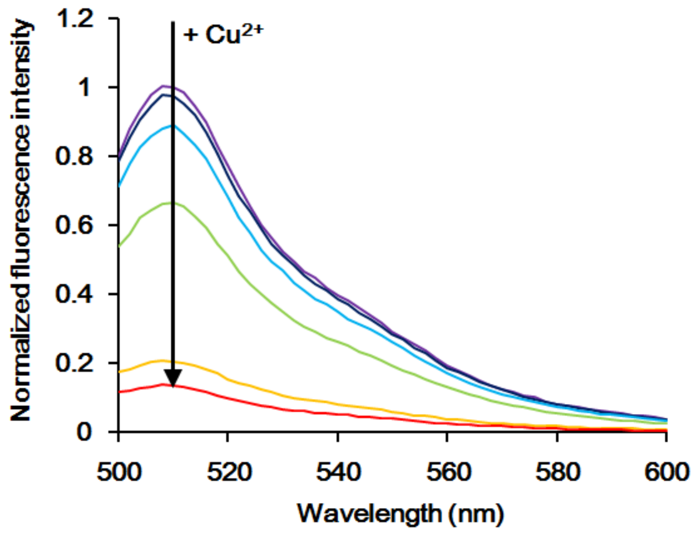

Fig. S14 Fluorescence responses of 1 μM GCS-2 after the addition of 0, 0.1, 0.2, 0.4, 0.8, and 1.6 μM Cu^{2+}. Fluorescence spectra were recorded within 5 min after Cu^{2+} addition.

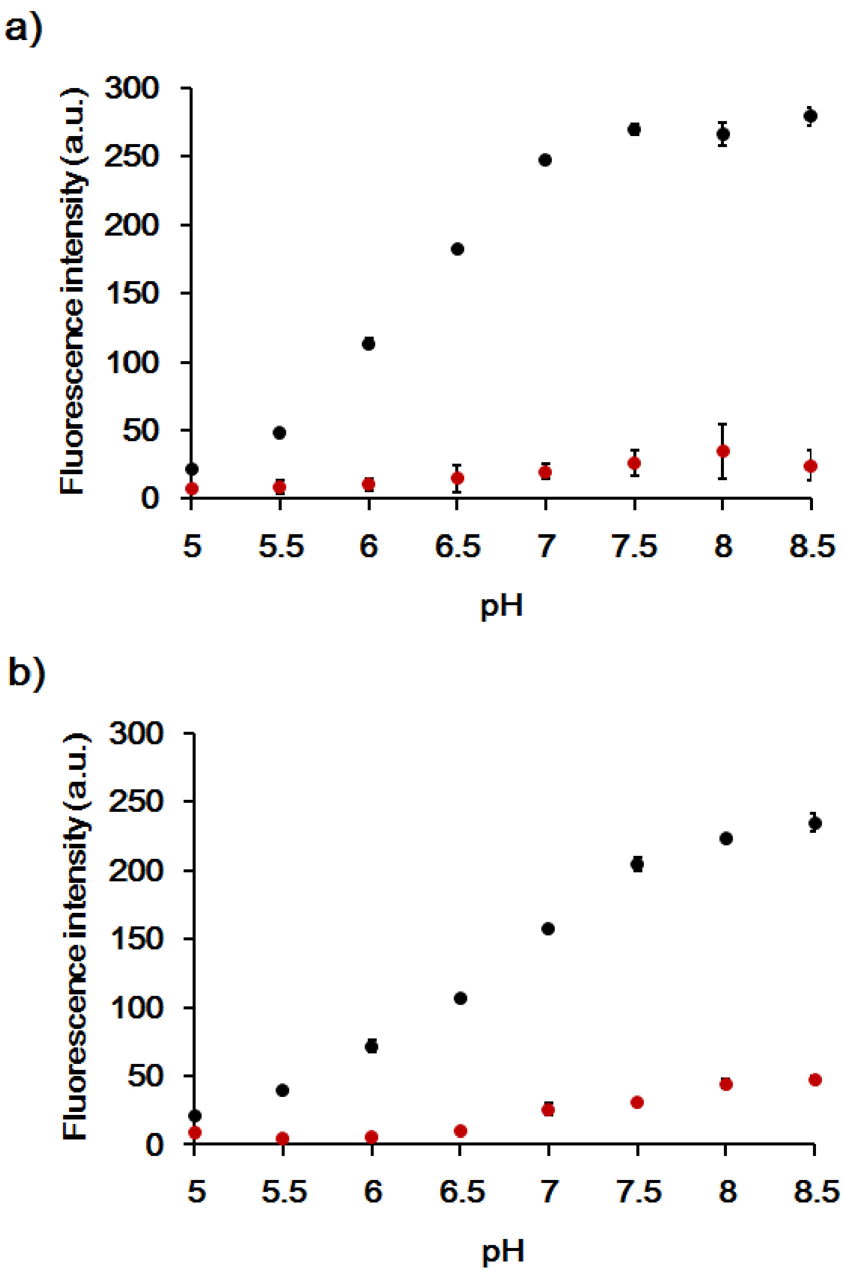

Fig. S15 pH-dependent fluorescence responses of a) GCS-1 and b) GCS-2 with (red dots) and without (black dots) Cu^{2+}. 1 μM proteins are mixed with 5 μM Cu^{2+}. The error bars correspond to the standard error of the mean of three independent measurements.

Supporting Table

Table S1. The apparent dissociation constants (K_D^{app}) for currently available genetically encoded Cu^{2+} sensors and GCS proteins.

Reporter Protein	K_D^{app} (μM)	Conditions	Reference
BFPms1	24	20 mM HEPES, pH 8.0	1
DsRed2	0.540 ± 0.090	10 mM MOPS, pH 7.2	2
	8.4 ± 1.2	100 mM HEPES, 300 mM NaCl, pH 7.0	this work
drFP583	14.80 ± 1.68	50 mM HEPES-KOH, 100 mM NaCl, 1 mM $MgCl_2$, 1 μg/mL BSA, pH 7.9	3
Rmu13	10.90 ± 1.74		
DsRed-Express	5.4 ± 1.7	20 mM MOPS, pH 7.4	4
DsRed-Monomer	1.7 ± 0.3		
GFPdopa	5.6 ± 0.3	20 mM MOPS, pH 7.4	5
GFP T203H with His_6 tag	9 ± 2	16 mM Na_2HPO_4, 4 mM KH_2PO_4, 115 mM NaCl, pH 7.4	6
wild-type EGFP	4.52 ± 0.77 (one site bind) 3.92 ± 0.73 (quadratic)	10 mM MOPS, pH 7.5	7
	65.6 ± 7.4	100 mM HEPES, 300 mM NaCl, pH 7.0	this work
EGFP S202H/Q204H	0.26 ± 0.025 (one site bind) 0.015 ± 0.009 (quadratic)	10 mM MOPS, pH 7.5	7
mEmerald-3H	0.2	3 mM HEPES, 130 mM NaCl, pH 7.4	8
GCS-2	0.00793 ± 0.00320	100 mM HEPES, 300 mM NaCl, pH 7.0	this work
GCS-1	0.09404 ± 0.01933		

Since values of K_D^{app} vary depending on experimental methods, dissociation constants for wild-type EGFP and DsRed2 which were prepared similarly to GCS proteins were determined as controls.

Experimental Methods

Chemicals

Copper (II) sulfate (Alfa Aesar, purity 98 %) was used for the copper (II) source for all *in vitro* measurements. Other salts including $ZnCl_2$, $NH_4Fe(SO_4)_2 \cdot 12H_2O$, $CoCl_2 \cdot 6H_2O$, $NiCl_2 \cdot 6H_2O$, $CdCl_2$, $AgNO_3$, $Cr(NO_3)_3 \cdot 9H_2O$, $Hg(NO_3)_2 \cdot 2H_2O$, and $Pb(NO_3)_2$ were also purchased from Alfa Aesar with purity >98 %. Copper (I) tetrakis(acetonitrile) hexafluorophosphate (97 %) was purchased from Sigma Aldrich. Calcium (II) chloride dihydrate, $MnCl_2 \cdot 4H_2O$, KCl, and $MgCl_2 \cdot 6H_2O$ were purchased from Junsei Chemical with purity >98 %. Copper (II) chloride dihydrate (Junsei, 97 %) was used for the copper (II) source for *in vivo* experiments.

Cloning of genetically encoded copper (II) sensor (GCS) proteins for bacterial expression

An insert containing the N-terminal amino terminal copper- and nickel-binding (ATCUN) motif and the circularly permuted OPT was constructed through two successive PCR reactions using a OPT β7-6 gene, subcloned from the synthesized OPT gene (Bioneer)[9]. For the first PCR, the primers OPT-8-fw (5'- CAA TGT ATA CAT CAC GGC AGA CAA ACA AAA GAA TGG AAT CAA AGC TAA C) and OPT-EcoRI-rv (5'- AAA *GAA TTC* TCA GTT GTA TTC GAG TTT GTG TCC; incorporates an *EcoRI* site) were used for the amplification. For the second PCR reaction for GCS-1, the first PCR product was used for the template, and the primers 2D-OPT-fw (5'- GGT GGT T*GC TCT TCC* AAC GGA GGA CAC AAT GTA TAC ATC ACG GCA G; incorporates the GGH sequence and a *SapI* site) and OPT-EcoRI-rv

were used. For GCS-2, +1-OPT-fw (5'- GGT GGT T*GC TCT TCC* AAC GGA GGA CAC CAC AAT GTA TAC ATC ACG GCA G; incorporates the GGH sequence and a *SapI* site) was used instead. The inserts were digested with SapI and EcoRI and ligated in-frame to the similarly digested pTYB21 vector, which introduces an N-terminal intein tag.

Cloning of GCS proteins for mammalian expression (GCS-1-TM and GCS-2-TM)

An insert containing a signal sequence for secretion, an ATCUN motif, and the circularly permuted OPT was constructed through overlap extension PCR. For the first fragment of GCS-1, the synthesized Apomab[10] gene (Bioneer) was used for the template, and the primers SS-EcoRI-fw (5'- TTT *GAA TTC* GCC ACC ATG GGC TGG AGC TGC ATC; incorporates a part of signal sequence and an *EcoRI* site) and SS-OE-rv (5'- GAT GTA TAC ATT GTG TCC TCC GCT GTG CAC GCC GG) were used for the amplification.[10] For the second fragment, the primers 2D-GGH-fw (5'- GGA GGA CAC AAT GTA TAC ATC; incorporates the GGH sequence) and 2D-O-PstI-rv (5'- AAA *CTG CAG* GTT GTA TTC GAG TTT GTG TCC; incorporates a *PstI* site) were used. For PCR reactions for GCS-2, primers SS+1-OE-rv (5'- GAT GTA TAC ATT GTG GTG TCC TCC GCT GTG CAC GCC GG) and SS+1-OE-fw (5'- GGA GGA CAC CAC AAT GTA TAC ATC; incorporates the GGH sequence) were used instead. Overlap extension PCR of the first and second fragment was performed using the primers SS-EcoRI-fw and 2D-O-PstI-rv to generate the final insert. The insert was digested with EcoRI and PstI and ligated in-frame to the similarly digested pDisplay vector, which introduces a C-terminal platelet-derived growth factor receptor (PDGFR) transmembrane domain.

Cloning of mCherry for mammalian expression (mCherry-TM)

An insert containing a signal sequence for secretion and mCherry was constructed through overlap extension PCR. For the first fragment, the synthesized Apomab gene was used for the template, and the primers SS-EcoRI-fw and SS-MC-OE-rv (5'- CCT CGC CCT TGC TCA CGC TGT GCA CGC CGG) were used for the amplification. For the second fragment, the synthesized mCherry gene (Bioneer) was utilized for the template, and the primers SS-MC-fw (5'- GTG AGC AAG GGC GAG G) and SS-MC-PstI-rv (5'- AAA *CTG CAG* CTT GTA CAG CTC GTC CAT G; incorporates a *PstI* site) were used. Overlap extension PCR of the first and second fragment was performed using the primers SS-EcoRI-fw and SS-MC-PstI-rv to generate the final insert. The insert was digested with EcoRI and PstI and ligated in-frame to the similarly digested pDisplay vector.

Expression and purification of GCS proteins

A pTYB21 plasmid containing the GCS gene was introduced into *E.coli* strain BL21 (DE3, HiT-21, RBC Bioscience) by heat-shock transformation. The cells were cultured in LB supplemented with ampicillin (100 μg/mL) at 37 °C until OD_{600} 0.4. The culture flask was then placed at 20 °C until OD_{600} 0.6. Protein expression was induced by the addition of IPTG to a final concentration of 1 mM. After incubation at 20 °C for >16 h, the cells were harvested by centrifugation. The cells were lysed by sonication on ice (7-second pulse at 40 % amplitude with 5 seconds between each pulse for 20 min) in column binding buffer (20 mM Tris pH 8.5, 500 mM NaCl). Purification of GCS proteins was performed using the Impact Kit (New England Biolabs) following the manufacturer's protocol. Briefly, the lysate was cleared by centrifugation, and the supernatant was loaded onto a chitin column. After

washing with 20 bed volumes of column binding buffer, the column was incubated in cleavage buffer (20 mM Tris pH 8.5, 500 mM NaCl, 50 mM DTT) at 25 ℃ overnight. Fractions containing the desired proteins were consolidated and dialyzed twice in a buffer containing 100 mM HEPES pH 7.0, and 300 mM NaCl. For measurements with Ag^{+} and Pb^{2+}, a fraction of protein was further dialyzed against a buffer containing 100 mM HEPES pH 7.0, and 300 mM $NaNO_3$. The size and purity of the GCS proteins were analyzed by 12 % SDS-PAGE. The concentration of protein was determined by Bradford assay (Bio-Rad).

N-terminal sequencing for a fusion protein of ATCUN and circularly permuted EGFP (ATCUN-cpEGFP)

The ATCUN-cpEGFP protein was generated and purified in a manner similar to that described above. After separation in a 12 % SDS-PAGE gel, the protein was transferred to a PVDF membrane and stained with 0.1 % Coomassie brilliant blue R-250 in 50 % methanol. The membrane was then washed several times with 50 % methanol and dried before submission for protein sequencing. N-terminal Edman sequencing of the sample was performed at Korea Basic Science Institute using an Applied Biosystems (ABI) Procise 492 protein sequencer.

Fluorescence measurement

Fluorescence spectra were obtained using either a Varioskan FLASH plate reader (Thermo Scientific) or FluoroMate FS-2 Fluorometer (Scinco). For Varioskan measurements, protein samples in black 96-well plates were excited at 480 nm (bandwidth = 5 nm), and emission

signals were recorded from 500 to 600 nm with a 2 nm step size. For measurements with FluoroMate (PMT voltage = 700 V, scan speed = 1200 nm/min, integration time = 20 ms, response time = 0.1 s), protein samples in transparent 2 mL cuvettes (path length = 1 cm) were excited at 480 nm (Ex slit = 5 nm), and emission signals were recorded from 500 to 600 nm (Em slit = 5 nm). Collected data were processed using MS Excel software. Unless stated otherwise, all fluorescence measurements were repeated three times, and average values were used for analysis.

Cu^{2+} titration experiment

To monitor fluorescence changes with increasing concentrations of Cu^{2+}, 1 μM solutions of GCS-1 or GCS-2 in assay buffer (100 mM HEPES pH 7.0, 300 mM NaCl) were treated with final concentrations of 0, 0.1, 0.2, 0.4, 0.8, 1.6, 3.2, and 6.4 μM $CuSO_4$. Fluorescence spectra were collected either within 5 min or after 1 h incubation at room temperature with a Varioskan fluorometer.

Comparison of fluorescence quenching properties of GCS proteins

To compare the fluorescence quenching profiles of the GCS and control proteins, 0.5 μM protein solutions in assay buffer were treated with final concentrations of 0, 1, 10, and 50 μM $CuSO_4$. Fluorescence spectra were collected within 5 min or after 1 h incubation at room temperature with a Varioskan fluorometer. Fluorescence signals at 510 nm were used for analysis. To monitor the fluorescence responses of the GCS and control proteins at lower concentrations of Cu^{2+}, 0.1 μM protein solutions in assay buffer were treated with final

concentrations of 0 and 0.5 μM $CuSO_4$. Fluorescence spectra were collected after 1 h incubation at room temperature with Fluoromate. Fluorescence signals at 510 nm were used for analysis.

Reversibility test for Cu^{2+}-bound GCS proteins

One micro molar solutions of GCS proteins in assay buffer were treated with final concentrations of 0 and 5 μM $CuSO_4$. Fluorescence spectra were collected within 5 min, and a final concentration of 50 μM ethylenediaminetetraacetic acid (EDTA) was added. After equilibration, fluorescence spectra were collected with a Varioskan fluorometer. Fluorescence signals at 510 nm were used for analysis.

Cu^{2+} selectivity measurement

To investigate the Cu^{2+} selectivity of GCS proteins, 1 μM solutions of GCS-1 or GCS-2 in assay buffer were treated with 5 μM of various metal ions including Cr^{3+}, Mn^{2+}, Fe^{3+}, Co^{2+}, Ni^{2+}, Zn^{2+}, Cd^{2+}, Hg^{2+}, K^{+}, Ca^{2+}, and Mg^{2+}. For measurement with Pb^{2+} and Ag^{+}, 1 μM solutions of GCS-1 or GCS-2 in a HEPES buffer (100 mM HEPES pH 7.0, 300 mM $NaNO_3$) were used. Stock solutions of metal ions were prepared at 100 μM in Millipore water. After incubation at room temperature for 1 h, fluorescence spectra were collected with a Varioskan fluorometer. Following the measurement, $CuSO_4$ was added to each well to a final concentration of 5 μM and incubated at room temperature for 1 h. Fluorescence spectra were again collected under the same conditions. Fluorescence signals at 510 nm were used for analysis, and relative fluorescence changes were plotted against each metal ion.

For the Cu^+ sensing experiment, 1 μM solutions of GCS-1 or GCS-2 in assay buffer including 250 μM ascorbic acid were treated with 5 μM Cu^+. The Cu^+ stock solution was prepared at 100 μM in acetonitrile. In a control experiment lacking any metal ion, acetonitrile was added. Fluorescence spectra were recorded as above within 5 min after Cu^+ addition.

Determination of Cu^{2+} binding constant

To determine the apparent Cu^{2+} binding constant for GCS-2, 2 mL of 50 nM GCS-2 including various concentration of $CuSO_4$ (0, 3.125 nM, 6.25 nM, 12.5 nM, 25 nM, 50 nM, 100 nM, 200 nM, 400 nM, 800 nM) were incubated at room temperature until they equilibrated. The fluorescence spectra were collected at room temperature using FluoroMate. Fluorescence signals at 510 nm were used to calculate the binding constant. As mentioned in the main text, we used Equation (1):

$$\frac{\Delta F}{\Delta F_{\mathrm{max}}} = \frac{\left(K_{\mathrm{D}}+[\mathrm{Cu}]+[\mathrm{P}]\pm\sqrt{(K_{\mathrm{D}}+[\mathrm{Cu}]+[\mathrm{P}])^2-4[\mathrm{Cu}][\mathrm{P}]}\right)}{2[\mathrm{P}]} \quad (1)$$

where, ΔF is the change in measured fluorescence, ΔF_{max} is the maximum fluorescence change, K_{D} is the dissociation constant of the sensor protein, [P] is the total protein concentration, and [Cu] is the total concentration of Cu^{2+}. The $\Delta F/\Delta F_{\mathrm{max}}$ values were plotted against copper concentration, and the curves were fitted using SigmaPlot software. The experiment was repeated three times. K_{d} values derived from each fitting result were averaged and reported with the standard error of the mean.

For GCS-1, experiments were performed as described above with 2 mL of 100 nM GCS-1 including 0, 12.5 nM, 25 nM, 50 nM, 100 nM, 200 nM, 400 nM, 800 nM, 1.6 μM, 3.2

μM $CuSO_4$.

Competition experiment with human serum albumin (HSA) and amyloid beta peptide (Aβ40)

Essentially fatty acid free HSA (Sigma) was used without further purification, and its concentration was measured using the molecular extinction coefficient at 280 nm (34445 M^{-1} cm^{-1}).[11] Approximately 15 % of HSA molecules have truncated N-termini; thus, they do not bind copper through ATCUN motifs.[12] To investigate the relative binding affinities of HSA and GCS proteins, 1 μM GCS-2 solutions in assay buffer were mixed with 0, 0.5, 1, and 5 μM HSA and then treated with final concentrations of 0, 0.2, 0.4, 0.6, 0.8, 1.0, 1.2, 1.4, 1.6, 1.8, 2.0, and 2.2 μM $CuSO_4$. For GCS-1, 1 μM GCS-1 solutions were mixed with 0 and 1 μM HSA and then treated with final concentrations of 0, 0.2, 0.4, 0.6, 0.8, 1.0, 1.2, 1.4, 1.6, 1.8, 2.0, and 2.2 μM $CuSO_4$. Protein samples were incubated at 25 °C for 24 h, and fluorescence spectra were measured with VarioSkan.

A stock solution of Aβ40 peptide was prepared as previously described.[13] Briefly, lyophilized Aβ40 peptides (Sigma) were dissolved in 1,1,1,3,3,3-hexafluoro-2-propanol (HFIP, Sigma) at a concentration of 500 μM to generate peptide monomers. HFIP was then removed by evaporation in a vacuum. The dried sample was re-suspended in HFIP, and the solvent was removed as before. Aβ-HFIP was dissolved in 0.02 % NH_3 solution at a concentration of 50 μM. The actual peptide concentration was determined to be 29.1 μM using the extinction coefficient at 280 nm, 1280 M^{-1} cm^{-1}.[14] The stock solution was maintained on ice and used for experiments within 24 h. To investigate the relative binding affinities of Aβ40 and GCS proteins, 1 μM solutions of GCS-1 or GCS-2 in assay buffer were

mixed with 0 and 1 μM Aβ40 and then treated with final concentrations of 0, 0.2, 0.4, 0.6, 0.8, 1.0, 1.2, 1.4, 1.6, 1.8, 2.0, and 2.2 μM $CuSO_4$. Protein samples were incubated at 25 °C for 24 h, and fluorescence spectra were measured with VarioSkan. Experiments were repeated twice, and averaged values were used for analysis.

UV-VIS Measurements

An absorption spectrum was recorded at room temperature using a UV-VIS spectrophotometer (Optizen, Mechasys). Two milliliters of 10 μM GCS-1 or GCS-2 in assay buffer was used for the measurement. Additionally, 10 μM protein solutions were treated with 20 μM $CuSO_4$ and observed under the same settings. The spectrum data were processed with Microsoft Excel.

pH dependency measurement

To investigate the pH sensitivity of GCS proteins, 1 μM solutions of GCS-1 or GCS-2 in various buffers (pH 5, 5.5, 6, and 6.5: 100 mM MES and 300 mM NaCl, pH 7, 7.5, 8, and 8.5: 100 mM HEPES and 300 mM NaCl) were treated with 5 μM $CuSO_4$. In a control experiment that lacked any metal ion, millipore water was added. After incubation at room temperature for 1 h, fluorescence spectra were collected with a Varioskan fluorometer. Fluorescence signals at 510 nm were plotted against the pH of buffer solutions.

Imaging GCS proteins on live HeLa cell surfaces

HeLa cells were maintained in Dulbecco's modified Eagle's medium (DMEM, Gibco) containing 10 % fetal bovine serum (FBS) and 1 % penicillin-streptomycin at 37 ℃ in 5 % CO_2. The day before transfection, 1.6-2×10^4 HeLa cells were seeded in a 96-well glass-bottom microplate (Matriplate). The cells were then transfected with the GCS-2-TM plasmid using Lipofectamine 2000 (Invitrogen). After 16-24 h, imaging was performed with a Nikon Ti total internal reflection fluorescence (TIRF) microscope using a Nikon Apochromat TIRF 60× (NA=1.49) or a Nikon Plan Apochromat 60× (NA=1.40) objective. Diode and a directly doubled diode (DDD) lasers (488 nm for GFP and 561 nm for mCherry, CVI-Melles Griot) were used for excitation, and the Nikon emission filters FITC HYQ (DM505, BA535/50) and G-2A (DM575, BA590) were used for the GFP and mCherry channels, respectively. GFP and mCherry images were captured by an EMCCD camera (C9100-02; Hamamatsu Photonics) and analyzed using NIS-element AR 64-bit version 4.1 (Laboratory Imaging) or MetaMorph version 7.7 (Molecular Devices, LLC) software. $CuCl_2$ solutions were prepared from an aqueous 1 mM stock solution by diluting with Dulbecco's phosphate-buffered saline (DPBS, -calcium, -magnesium, Gibco). EDTA solutions were prepared from an aqueous 500 mM stock solution. Prior to data collection, the cells were rinsed once with 200 μL DPBS and then incubated with 100 μL DPBS. Multiple positions per well were selected for each imaging experiment. Images were collected prior to the addition of copper, and 100 μL of 100 μM $CuCl_2$ (the final concentration of copper was 50 μM) was added to the cells. Images were collected every 30 s in a 4-min period. Subsequently, EDTA was added to a final concentration of 1 mM, and responses were observed every 30 s for 4 min. Responses to zinc were also examined by adding 50 μM $ZnCl_2$ followed by 50 μM $CuCl_2$.

For control experiments with mCherry, HeLa cells were co-transfected with the GCS-2-TM and mCherry-TM plasmids as described above. Images were acquired prior to

copper addition, and 100 μL of 20 μM $CuCl_2$ was added to the cells. Images were collected every 30 s for 4 min. To acquire DIC images, a confocal microscope (Nikon Ti-E) equipped with a CFI Plan Apochromat VC 60× (NA=1.40) objective and DIC detector was used. HeLa cells with surface-expressed cpOPT were similarly studied.

For quantitative analysis of fluorescence responses during live-cell imaging, regions of interest (ROIs) were drawn for cellular responses at each position. To perform background subtraction, regions where no cells were present were randomly selected for background ROIs at each position. The intensity of the background was subtracted from the fluorescence intensity of the cellular response and normalized to that of a no metal ion control.

References

1. D. P. Barondeau, C. J. Kassmann, J. A. Tainer and E. D. Getzoff, *J. Am. Chem. Soc.*, 2002, **124**, 3522-3524.
2. J. P. Sumner, N. M. Westerberg, A. K. Stoddard, T. K. Hurst, M. Cramer, R. B. Thompson, C. A. Fierke and R. Kopelman, *Biosens. Bioelectron.*, 2006, **21**, 1302-1308.
3. P. Eli and A. Chakrabartty, *Protein Sci.*, 2006, **15**, 2442-2447.
4. Y. Rahimi, A. Goulding, S. Shrestha, S. Mirpuri and S. K. Deo, *Biochem. Biophys. Res. Commun.*, 2008, **370**, 57-61.
5. N. Ayyadurai, N. S. Prabhu, K. Deepankumar, S.-G. Lee, H.-H. Jeong, C.-S. Lee and H. Yun, *Angew. Chem. Int. Ed.*, 2011, **50**, 6534-6537.
6. B. Hotzer, R. Ivanov, S. Altmeier, R. Kappi and G. Jung, *J. Fluoresc.*, 2011, **21**, 2143-2153.

7. E. É. Bálint, J. Petres, M. Szabó, C. K. Orbán, L. Szilágyi and B. Ábrahám, *J. Fluoresc.*, 2013, **23**, 273-281.

8. X. Yu, M. P. Strub, T. J. Barnard, N. Noinaj, G. Piszczek, S. K. Buchanan and J. W. Taraska, *PLoS One*, 2014, **9**, e95808.

9. (a) S. Cabantous, T. C. Terwilliger and G. S. Waldo, *Nat. Biotechnol.*, 2005, **23**, 102-107; (b) J. D. Pedelacq, S. Cabantous, T. Tran, T. C. Terwilliger and G. S. Waldo, *Nat. Biotechnol.*, 2006, **24**, 79-88.

10. D. R. Camidge, *Expert Opin. Biol. Ther.*, 2008, **8**, 1167-1176.

11. C. N. Pace, F. Vajdos, L. Fee, G. Grimsley and T. Gray, *Protein Sci.*, 1995, **4**, 2411-2423.

12. B. Chan, N. Dodsworth, J. Woodrow, A. Tucker and R. Harris, *Eur. J. Biochem.*, 1995, **227**, 524-528.

13. V. Tougu, A. Karafin and P. Palumaa, *J. Neurochem.*, 2008, **104**, 1249-1259.

14. O. Crescenzi, S. Tomaselli, R. Guerrini, S. Salvadori, A. M. D'Ursi, P. A. Temussi and D. Picone, *Eur. J. Biochem.*, 2002, **269**, 5642-5648.
